# Genomics and resequencing of *Fagopyrum dibotrys* from different geographic regions reveals species evolution and genetic diversity

**DOI:** 10.3389/fpls.2024.1380157

**Published:** 2024-06-11

**Authors:** Si-hao Zheng, Yong-chao Diao, Jie Du, Jin-tong Li, Sha Zhao, Mei-juan Liu, Hui-cai Lin, Yan Zeng, Ji-yong Wang

**Affiliations:** ^1^ China National Traditional Chinese Medicine Co., Ltd, Beijing, China; ^2^ China Traditional Chinese Medicine Seed&Seeding, Co., Ltd, Beijing, China

**Keywords:** genomics_1_, resequencing_2_, *Fagopyrum dibotrys*_3_, evolution_4_, genetic diversity_5_

## Abstract

*Fagopyrum dibotrys*, belonging to the family *Polygonaceae* and genus *Fagopyrum*, is used in traditional Chinese medicine and is rich in beneficial components, such as flavonoids. As its abundant medicinal value has become increasingly recognized, its excessive development poses a considerable challenge to wild germplasm resources, necessitating artificial cultivation and domestication. Considering these factors, a high-quality genome of *F. dibotrys* was assembled and the evolutionary relationships within *Caryophyllales* were compared, based on which 58 individual samples of *F. dibotrys* were re-sequenced. We found that the samples could be categorized into three purebred populations and regions distributed at distinct elevations. Our varieties were cultivated from the parental populations of the subpopulation in central Yunnan. *F. dibotrys* is speculated to have originated in the high-altitude Tibetan Plateau region, and that its combination with flavonoids can protect plants against ultraviolet radiation; this infers a subpopulation with a high accumulation of flavonoids. This study assembled a high-quality genome and provided a theoretical foundation for the future introduction, domestication, and development of cultivated varieties of *F. dibotrys*.

## 1 Introduction

*Fagopyrum dibotrys*, commonly known as golden buckwheat, belongs to the genus *Fagopyrum* and the *Polygonaceae* family. Genus *Fagopyrum* presently comprises 15 species, including *F. tataricum*, *F. esculentum*, *F. dibotrys*, etc. The compounds contained in the genus *Fagopyrum* can be divided into six classes: flavonoids, phenolics, fagopyritols, triterpenoids, steroids, and fatty acids (Jing et al., 2016). Flavonoids represent a substantial accumulation in the genus *Fagopyrum*, especially six major classes: quercetin, rutin, orientin, homoorientin, vitexin, and isovitexin (Joshi et al., 2020). Flavonoids can also protect plants from environmental pressure and against biotic stressors (Shen et al., 2022). Among these, rutin and quercetin are highly accumulated in *F. dibotrys*. Rutin can effectively protect against cardiovascular and cerebrovascular diseases and liver damage (Lee et al., 2013), quercetin can prevent several ailments, including cardiovascular diseases, cancer, and tumors (Xu et al., 2019), and the rhizome of *F. dibotrys* is the most abundant and primary medicinal part of the plant. In *F. dibotrys*, although the types of flavonoid compounds are relatively clear, the pathways of flavonoid biosynthesis have not yet been fully elucidated. The biosynthesis of flavonoids in plants involves several enzymatic steps. Phenylalanine is produced by the shikimate pathway and then converted to cinnamic acid through the action of phenylalanine ammonialyase (PAL). Cinnamic acid is hydroxylated at the C4 position by cinnamic acid hydroxylase (C4H) to form p-coumaric acid, which is then catalyzed into p-coumaroyl CoA by coumarin CoA ligase (4CL). Chalcone synthase (CHS) catalyzes the condensation and isomerization of p-coumaroyl CoA to produce naringenin chalcone, which is then converted to chalcone by chalcone isomerase (CHI). Naringenin undergoes hydroxylation at various positions by flavanone 3-hydroxylase (F3H), flavonoid 3’-hydroxylase (F3’H), and flavonoid 3’,5’-hydroxylase (F3’5’H) to produce dihydroquercetin. Dihydroquercetin can then be further converted into rutin, quercitrin, epicatechin, catechin, and kaempferol through a series of complex reactions (Huang et al., 2022; Shen et al., 2022).

In China, this plant is classified as a second-level protected plant. Throughout history, *Fagopyrum* is considered to have originated in China, particularly in the southwestern regions bordering the provinces of Yunnan, Sichuan, and Tibet (Hunt et al., 2018). *Fagopyrum* is distributed throughout countries around the Himalayan Mountains. Unlike *F. dibotrys*, *F. tataricum*, and *F. esculentum* are often used as staple crops for human consumption. *F. dibotrys* has long been used in China as a traditional medicinal herb because of its rich array of valuable bioactive constituents such as flavonoids and phenolics. The rhizome is a crucial component of Chinese herbal medicines. During the Qing Dynasty, Bencaoshiyi reported that the rhizome could be used to treat lung ailments, rheumatism, and abdominal pain. The extract from the rhizome of *F. dibotrys* inhibits the growth of lung, liver, colon, leukocyte, and bone cancer cells (Chan, 2003), and serves as the main active ingredient in lung cancer treatment drugs known as “WeiMaiNing” capsules (Ke et al., 2021). As research on *F. dibotrys* continues to progress, its value and various uses are gradually being discovered and applied. Simultaneously, the increasing demand has led to increased attention towards the exploitation of wild germplasm resources. This process not only exacerbates the degradation of the ecology and environment but also results in the overexploitation of wild germplasm resources, causing a rapid decline in population numbers in a short period and disrupting the local ecological balance and diversity of the species. Therefore, the rational and efficient utilization of wild germplasm resources for the domestication and breeding of cultivated varieties through artificial reproduction methods is crucial for addressing the current excessive demand. Obtaining a high-quality genome of *F. dibotrys* may not only provide insights into the evolution of the genus *Fagopyrum* but also establish foundational data for resequencing and mining genes involved in flavonol biosynthesis. This necessitates an understanding of the distinctions between *F. dibotrys* varieties in different regions. Therefore, understanding the evolutionary relationships and genetic backgrounds of different subpopulations is necessary. The purposeful selection of superior subpopulations for hybridization and cultivation of cultivated varieties is imperative, as it will guide breeding efforts in developing cultivated varieties to meet the demands of the vast market.

In the present study, a high-quality *F. dibotrys* genome is reported. Additionally, its evolutionary relationships within the *Caryophyllales* order were explored and 46 wild samples from different geographical locations and 12 cultivated varieties were re-sequenced. These re-sequenced samples can be divided into subpopulations based on the genetic differences identified through population evolutionary analysis and genetic foundation. The potential origin of *F. dibotrys* and the subpopulation with the highest flavonoid content was inferred. Furthermore, the limitations of the introduction and domestication were identified. The resequencing of samples from different regions can reveal their genetic backgrounds, facilitating targeted breeding efforts for cultivated varieties. This will facilitate the cultivation of superior and high-yielding *F. dibotrys* varieties to meet the demands of an extensive market. In addition, this study contributes to the conservation of wild germplasm resources.

## 2 Materials and methods

### 2.1 Plant material acquisition and DNA extraction

The representative *F. dibotrys* individuals used were obtained from the plantation located in Xiaoshao Village, Dabanqiao Town, Guandu District, Kunming City, Yunnan Province located at 25°11′ 8.2176″ N, 102°58′ 54.7032″ E and an altitude of 1982 meters above sea level, China. Fresh, healthy mature leaves were collected for high-quality PacBio HiFi and Hi-C library construction. In addition, re-sequenced individual samples were collected from various regions in Yunnan, Guizhou, Chongqing, and Sichuan provinces. We also collected one seed-propagated and several cultivated varieties (**Supplementary Table S1**). After harvesting, all materials were immediately frozen in liquid N_2_. High-quality genomic DNA was extracted using the E.Z.N.A. Tissue DNA kit (Omega Bio-Tek) following the Tissue DNA-Spin Protocol. Subsequently, their quality was tested, and the DNA samples with yield of > 3 μg, concentration of >30 μg/μL, and OD_260_/OD_280_ 1.80–2.00 were qualified for use in further study.

### 2.2 Genome survey and sequencing

Before whole-genome sequencing, a 450 bp paired-end read library was constructed, and *k*-mer frequency distribution was used to estimate the genome size of *F. dibotrys*. First, the DNA was fragmented to ~ 450 bp using a Covaris M220, and T4 DNA polymerase was applied to generate blunt ends. After adding an ‘A’ base to the 3’ end of the blunt phosphorylated DNA fragments, adapters were ligated to the ends of the DNA fragments. Second, the desired fragments were purified by gel electrophoresis, selectively enriched, and amplified by PCR. The index tag was introduced into the adapter at the PCR stage, followed by library quality testing. Finally, paired-end libraries were quantified using the BGI MGISEQ-2000 platform. FastQC (https://www.bioinformatics.babraham.ac.uk/projects/fastqc/) with default parameters was used to assess sequencing data quality. Trimmomatic (Bolger et al., 2014) with default parameters was used to filter the short reads. Then, *k*-mers were counted using KMC (https://github.com/tbenavi1/KMC) with the parameters k27, ci1, and cs50000. The output file was analyzed using Smudeplot (Ranallo-Benavidez et al., 2020) and GenomeScope2 (https://github.com/tbenavi1/genomescope2.0) with default parameters to estimate ploidy and genome size. Genomic DNA samples were subsequently fragmented using Megaruptor, and high-quality fragments in the 13–16 kb range were selected using Sage ELF. The DNA library was constructed using SMRTbell following the protocol of the PacBio Sequel II platform. Circular consensus sequencing (CCS) was performed to obtain HiFi reads, and the lengths of subreads were subsequently analyzed, the visualization was performed using the ggplot2 (https://ggplot2.tidyverse.org/) package.

The same part of the plant was used to construct the Hi-C library, and 1% formaldehyde was used to fix young leaves for crosslinking. A Dounce homogenizer was used to lyse the cells, and MboI was used to digest the DNA. The ends of the DNA fragments were incorporated into adapter sequences containing biotin-labeled bases and ligated using sticky ends. The products were purified, ultrasonicated, and re-fragmented; only biotin-labeled pieces were captured and used to construct Illumina sequencing libraries (Belton et al., 2012). The DNA library was interrupted by high-frequency ultrasonication for resequencing, resulting in fragments of approximately 450 bp in length, following the Illumina standard protocol. Paired-end reads (2 × 150 bp) were sequenced using an Illumina Hiseq platform.

### 2.3 Genome assembly and quality assessment

The corrected CCS HiFi reads were assembled using HiFiasm V0.15.1 (Cheng et al., 2021, Cheng et al, 2022) with the following parameters: -l 3 -i –h1 –h2. Redundant sequences of the primary contigs were eliminated using purge haplotigs (Roach et al., 2018) with default parameters. Minimap2 V2.17 (Li, 2021) was used to align the HiFi reads to the assembled genome using the default parameters. The output data were sorted using SAMtools (Danecek et al., 2021). Next, MarkDuplicates software (https://gatk.broadinstitute.org/hc/en-us/articles/360037052812-MarkDuplicates-Picard) was used to remove duplicates with default parameters. Then BamDeal V0.24 (https://github.com/BGI-shenzhen/BamDeal) was used to calculate sequencing depth, GC ratio and depth, which were visualized using the gglot2 package. Finally, BUSCO v5.1.2 (Manni et al., 2021a, Manni et al., 2021b) was used to evaluate their completeness based on the embryophyta_odb10 library.

### 2.4 Hi-C-assisted genome assembly

Hi-C libraries were used to scaffold chromosomes and improve the accuracy of *F. dibotrys* genomic data. The Hi-C reads were filtered using SOAPnuke V1.6.5 (Chen et al., 2018) with parameters: filter -n 0.1 -l 15 -q 0.4 -A 0.25 –cutAdaptor -Q 2 -G –minLen 150. Juicer (Durand et al., 2016) was used to align the paired-end reads to the assembled genome and obtain effective Hi-C data (Hi-C Contacts). Thereafter, 3D-DNA (Dudchenko et al., 2017) was used to construct interaction matrices and obtain pseudochromosome-level scaffolds. The Hi-C data were divided into 300 Kb/bin, and a Hi-C interaction heatmap was generated. Completeness of the assemblies was evaluated using BUSCO (Manni et al., 2021b, Manni et al., 2021a) with the embryophyta_odb10 library. LAI (Ou et al., 2018) was used to determine genome completeness using LTR_retriever V1.9 (Ou & Jiang, 2018).

### 2.5 Genome annotation

A combination of homologous and *de novo* approaches was used to improve the identification of repeat sequences. First, RepeatModeler (https://www.repeatmasker.org/RepeatModeler/) and LTR_Finder (Xu & Wang, 2007) were used to build the *de novo* repetition library. RepeatMasker V4.0.7 (https://www.repeatmasker.org/RepeatMasker/) was used to identify repeat sequences based on the library and output data were used to generate four types of TE divergence plots using ggplot2. Next, the homolog method was then applied using the RepBase V21.12 (https://www.girinst.org/repbase/), RepeatMasker V4.0.7 (Benson, 1999), and RepeatProteinMask V4.0.7 (https://www.repeatmasker.org/cgi-bin/RepeatProteinMaskRequest) databases with default parameters to search for homologous repeat sequences. Tandem Repeats Finder V4.10.0 (Benson, 1999) was used to identify the tandem repeat sequences. Finally, the outputs of all the software were integrated and used as the final results.

Both *de novo* and homology-based methods were used for gene structure annotation. In the initial round of homology-based prediction methods, protein sequences from four species *Arabidopsis thaliana* (Cheng et al., 2017), *Beta vulgaris* (McGrath et al., 2023), *Fagopyrum tataricum* (Zhang et al., 2017), and *Solanum tuberosum* (Pham et al., 2020) were downloaded from the Phytozome (https://phytozome-next.jgi.doe.gov/) database and the China National Central for Bioinformation (https://ngdc.cncb.ac.cn/). Subsequently, approximately 3000 candidate genes obtained from this prediction were selected as gene models to train AUGUSTUS (Stanke et al., 2006). Subsequently, the model was trained for annotation using protein sequences from closely related species as input files in Maker (Holt & Yandell, 2011) with default parameters. Final consensus predictions were obtained through integration and deduplication using Maker (Holt & Yandell, 2011). BUSCO (Manni et al., 2021a, Manni et al., 2021b) was used to evaluate the completeness of the gene sets based on the embryophyta_odb10 library. For the functional annotation, BLAST V2.2.31 (Altschul et al., 1990) was chosen based on Non-Redundant Protein Database, InterPro (Apweiler et al., 2001) (https://www.ebi.ac.uk/interpro/), KEGG (Kanehisa & Goto, 2000) (https://www.genome.jp/kegg/), SwissProt (Boeckmann et al., 2003) (https://www.uniprot.org/), and KOG databases with default parameters.

**Figure 1:**
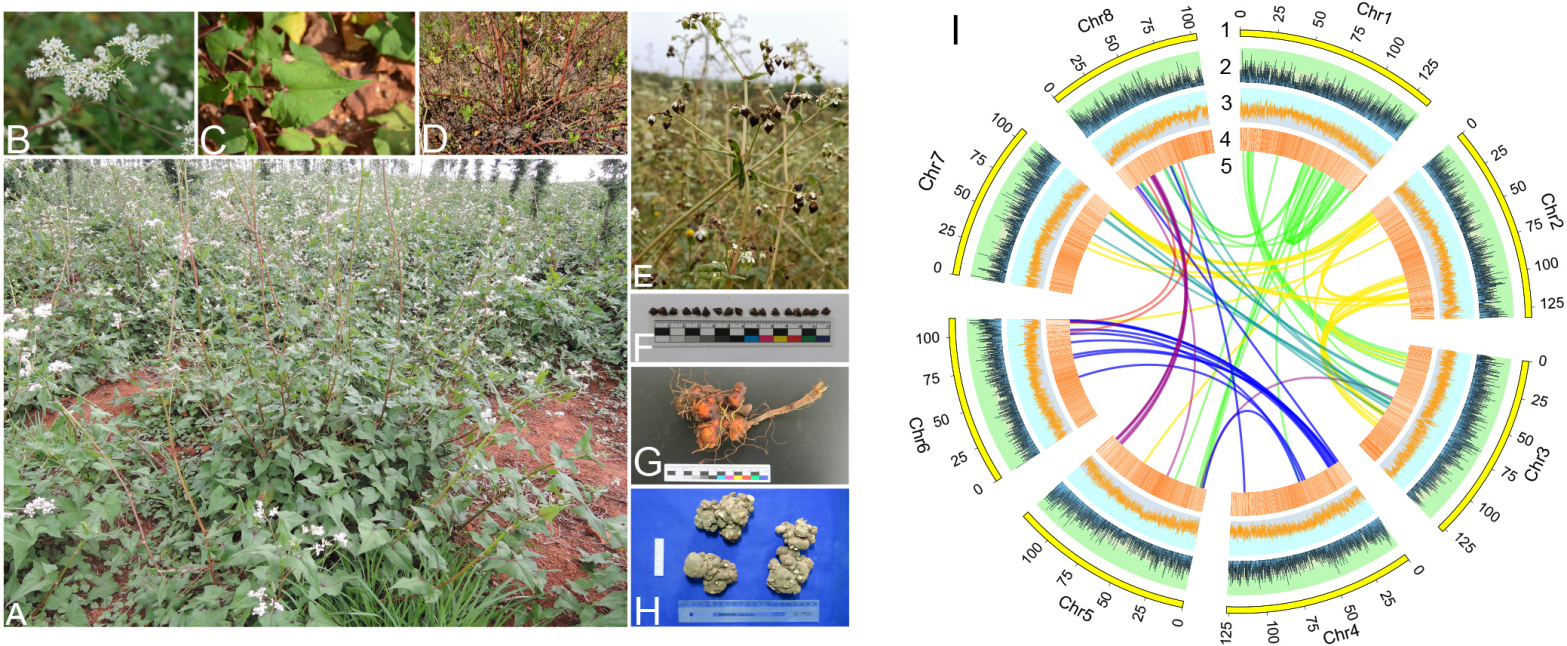
Morphology and genetic features of *F*. *dibotrys*. **(A)** Overall growth posture. **(B)** Inflorescence. **(C)** Leaves. **(D)** Stems. **(E, F)** Seeds. **(G, H)** Rhizomes. **(I)** Genomic landscape. (1–5): The lengths, gene count, GC count, percentage of repetitive sequences, and syntenic blocks of the eight pseudochromosomes.

For noncoding RNAs (ncRNAs), tRNAscan-SE V1.3.1 (http://lowelab.ucsc.edu/tRNAscan-SE/) (Lowe & Eddy, 1997) with default parameters was utilized for tRNA annotation. The rRNA fragments were predicted using BLAST with default parameters. The Rfam model in INFEANAL software (http://eddylab.org/infernal/) with default parameters was used to predict miRNAs and snRNAs. Based on the annotation results, we divided the assembled chromosome data into 50 kb windows to calculate gene counts, GC counts, and the percentage of repetitive sequences. We also included the syntenic blocks of the eight pseudochromosomes and presented them alongside the images of the plant features in **Figure 1**.

### 2.6 Phylogenetic and gene family analyses

To elucidate the evolutionary history of *F. dibotrys*, we selected eight additional species from the *Caryophyllales* order: *F. tataricum* (He et al., 2023), *F. esculentum* (He et al., 2023), *Portulaca amilis* (Gilman et al., 2022), *Gypsophila paniculata* (Li et al., 2022), *Amaranthus hypochondriacus* (Sunil et al., 2014), *Suaeda glauca* (Yi et al., 2022), *Spinacia oleracea* (Cai et al., 2021), and *Beta vulgaris* (McGrath et al., 2023), while *Vitis vinifera* (Olivier et al., 2007) was selected as an outgroup. OrthoFinder (https://github.com/davidemms/OrthoFinder/releases) was used to classify the gene families and identify single-copy families with the parameters -M msa -S diamond -T fasttree. All single-copy homologous genes were extracted and aligned using Muscle (http://www.drive5.com/muscle/) with the default parameters. These genes were selected to construct a phylogenetic tree using IQtree (Nguyen et al., 2015; Kalyaanamoorthy et al., 2017), treating *V. vinifera* as the outgroup, and the most appropriate model with the parameter (-m JTT+F+R4 -bb 1000) was selected. The divergence time was inferred using MCMCTree software (http://evolution.genetics.washington.edu/phylip.html) in the PAML (Mistry et al., 2021) package and calibrated using the TimeTree database (https://www.timetree.org). Subsequently, CAFE (Mendes et al., 2020) was used with default parameters to identify the contractions and expansions of gene families following the phylogenetic tree using a probabilistic graphical model of predicted divergence. Next, eggNOG-mapper (Hyatt et al., 2010; Eddy, 2011; Steinegger & Söding, 2017; Huerta-Cepas et al., 2019; Buchfink et al., 2021; Cantalapiedra et al., 2021) was used for the functional annotation of genes. Based on these results, KEGG pathway classification and enrichment analysis for significantly expanded gene families (p < 0.05) were conducted using clusterProfiler (Wu et al., 2021).

### 2.7 Whole-genome duplication and collinearity analysis

*F. dibotrys*, *F. esculentum* (He et al., 2023), *F. tataricum* (He et al., 2023), and *V. vinifera* (Olivier et al., 2007) were selected to identify recent whole genome duplication (WGD) events and collinearity relationships. WGD V1.2 (Chen & Zwaenepoel, 2023) (https://github.com/arzwa/wgd) was used to identify the most recent WGD events by analyzing the distribution of *K_s_* within paralogs with data visualization using the ggplot2 package. Subsequently, JCVI (https://github.com/tanghaibao/jcvi/wiki/MCscan-[Python-version]), with default parameters, was used to identify the collinearity blocks between these species. A collinearity block with at least five collinear gene pairs was constructed. Finally, CIRCOS V.0.69.8 (http://circos.ca/) software was used to visualize the gene count, gene synteny, percentage of repetitive sequences, and GC contents of individual pseudochromosomes.

### 2.8 RNA-seq data analysis

RNA-seq data was obtained from the NCBI SRA database (https://www.ncbi.nlm.nih.gov/Traces/study/?acc=SRP347690&o=acc_s%3Aa). During the withering stage and discontinuous removal of inflorescences in *F. dibotrys*, the following four tissue types of samples were selected: rhizome (RH), the top part of the stem (TS), the medium part of the stem (MS), and the basal part of the stem (BS); three biological replicates were set for each sample, totaling 12 transcriptome datasets.

The prefetch tool on the SRA Toolkit (https://github.com/ncbi/sra-tools/wiki/01.-Downloading-SRA-Toolkit) was used to download the raw data from the SRA database. Then, the fastq-dump of the sra-tools software (https://github.com/ncbi/sra-tools) was used to convert the downloaded data into the fastq format. The quality of the output files was evaluated using the FastQC software. Based on the FastQC results, the adapter sequences were trimmed using the Trimmomatic tool (https://github.com/usadellab/Trimmomatic/). HISAT2 (Kim et al., 2019) (http://daehwankimlab.github.io/hisat2/) was used to align clean reads to the reference genome using the parameters –gzip -split3. Uniquely mapped reads were retained according to the NH:i:1 tag in the HISAT2 BAM file. The SAM file generated after the alignment was converted into a BAM file by SAMtools (Danecek et al., 2021). The featureCount tool in the Subread software (https://subread.sourceforge.net/) was used to calculate read counts, and the edgeR package (Robinson et al., 2010) was used to calculate the FPKM value.

BLAST with default parameters was used to identify homologous sequences in the *F. dibotrys* genome, based on genes related to flavonoid biosynthesis in the GenBank database. Additionally, HMMER (http://hmmer.org/) was used to identify *CYP* and *UGT* genes related to flavonoid biosynthesis in *F. dibotrys* genome based on the Pfam database (CYP: PF00067, UGT: PF00201). Subsequently, multiple sequence alignment was performed using MAFFT (https://mafft.cbrc.jp/alignment/software/linux.html) with the parameter: -maxiterate 1000 –auto, and the resulting alignment was trimmed using trimAl (https://vicfero.github.io/trimal/) with the parameters -automated1, which was then used as input for IQtree (Nguyen et al., 2015) to construct the phylogenetic tree for further screening of candidate genes with the parameter: -m MFP -bb 1000. Finally, heatmaps based on FPKM values were generated using the pheatmap package (https://www.rdocumentation.org/packages/pheatmap/versions/1.0.12/topics/pheatmap). *Rheum tanguticum* (Li et al., 2023) was used for the comparative analysis. We constructed *CYP* genes and *UGT* genes phylogenetic trees of the other nine species selected for the species phylogenetic tree by FastTree with default parameters, and then IQtree was used to construct the phylogenetic trees based on the identified *F3’H*, *F3’5’H*, *RT*, and *UGT78D* genes with parameters: -m MFP -bb 1000.

### 2.9 MADS-box gene family analysis

MADS-box genes play crucial roles in almost all aspects of plant development. They play important regulatory roles in plant growth and development. Arabidopsis thaliana genes were used as query sequences to identify MADS-box genes in *F. dibotrys*, *F. esculentum*, and *F. tataricum* (He et al., 2023); the *Arabidopsis thaliana* genes were used as the query sequence. HMMER was used to identify MADS-box-related genes based on the PlantTFDB database (https://planttfdb.gao-lab.org/aboutus.php) with default parameters. Phylogenetic trees were constructed using IQtree with the parameter: -m MFP -bb 1000 (Nguyen et al., 2015). At last, MG2C (http://mg2c.iask.in/mg2c_v2.0/) was used to annotate and visualize the chromosome distribution. For further analysis of MADS-box in *F*. *dibotrys* genome, the online program GSDS 2.0 (http://gsds.cbi.pku.edu.cn/index.php) was used to map the gene structures. Bedtools (https://bedtools.readthedocs.io/en/latest/index.html) was used to obtain the upstream 2000 bp sequences as promoter regions, and the PlantCARE database (https://bioinformatics.psb.ugent.be/webtools/plantcare/html/) was used to predict cis-elements in their promoters. The MEME online programwas (https://meme-suite.org/meme/tools/meme) used to predict conserved motifs with a parameter of 10.

### 2.10 Resequencing library preparation and quality control

For Illumina pair-end sequencing, each sample containing at least 1 μg genomic DNA was used for sequencing library construction. Next, Covaris M220 was used to fragment the DNA into ~ 450 bp long pieces, following the standard genomic DNA library preparation protocol of the Illumina TruSeq™ Nano DNA Sample Prep Kit. Subsequently, a polyA tail was added to the 3’ end of the blunt phosphorylated DNA fragments, and adapters were ligated at both ends. The desired fragments were purified by gel electrophoresis, selectively enriched, and amplified by PCR. An index tag was introduced into the adapter at the PCR stage as required, followed by a library quality test. Finally, the quantified paired-end libraries were sequenced using the Illumina HiSeq platform (2 × 150 bp), and FastQC was used to evaluate the quality of the sequence reads. Trimmomatic was used to trim the raw data, and Burrows-Wheeler Aligner software (BWA) with the following parameters: bwa mem -k 32 (https://bio-bwa.sourceforge.net/index.shtml) was used to map the clean reads to the assembled reference genome and obtain the sequencing depth of the resequenced samples. Duplicate reads produced by PCR were removed using Picard tools (https://broadinstitute.github.io/picard/).

### 2.11 Variation detection and annotation

A valid BAM file was used to detect SNPs and short InDels (insertions and deletions) using the GATK HaplotypeCaller tool in the GATK toolkit (https://gatk.broadinstitute.org/hc/en-us) based on the Bayesian model, which can filter and refine the results for more accurate annotation. Variant call format (VCF) files were generated by quality filtering using VCF tools (https://vcftools.github.io/index.html) with the following parameters: QD < 2.0 || FS > 60.0 || MQ < 40.0 || SOR > 10.0. Subsequently, ANNOVAR (https://annovar.openbioinformatics.org/en/latest/user-guide/download/) was used to functionally annotate detected gene variants.

### 2.12 Population genetics

To explore genetic variations among different individuals, SNP annotation results from the resequenced individuals were used to construct an evolutionary tree using FastTree v2.1.10 (http://www.microbesonline.org/fasttree/), based on the neighbor-joining method. Thereafter, Principal Component Analysis (PCA) was used to classify the different subpopulations using GCTA v1.93.2 (https://yanglab.westlake.edu.cn/software/gcta/#Overview). Furthermore, population structure analysis was combined to ascertain the number of subpopulations among the resequenced individuals using fastSTRUCTURE (Raj et al., 2014) software with default parameters, and the different subpopulations were colored according to the clusters identified using fastSTRUCTURE software.

## 3 Result

### 3.1 Genome sequencing, assembly, and annotation

Genome size and ploidy were estimated using *k*-mer with short reads, revealing that *F. dibotrys* was a heterozygous diploid (**Supplementary Figure S1**). The estimated genome size was approximately 800 Mb with a heterozygosity of 1.4% (**Supplementary Figure S2**). Analyses of the GC ratio and depth (**Supplementary Figure S3**) showed that the sequenced samples were not polluted. Initially, a 314.36 Gb raw database was obtained using the PacBio Sequel II platform. Finally, we obtained 18.15 Gb of CCS data comprising 1.6 M HiFi reads, and ~ 21.81 × coverage of *F. dibotrys* (**Supplementary Table S2**), the sequencing depth was primarily between 8- to 27-fold (**Supplementary Figure S4**), and most reads were ~ 10 Kb in length (**Supplementary Figure S5**). After initial assembly and removal of duplicate sequences, the preliminary assembly results showed that the N50 value was 2.47 Mb and the N90 value was 735.20 kb (**Supplementary Table S3**).

To obtain a more accurate chromosome-level assembly, 142.77 Gb of high-quality Hi-C paired-end reads were generated, and the Q20 and Q30 values were 97.72% and 93.67%, respectively (**Supplementary Table S4**). Juicer results showed that 68 M Hi-C Contacts were obtained (**Supplementary Table S5**). After anchoring the scaffold with Hi-C sequencing data, we obtained 967.5 Mb of high-quality data spanning eight pseudochromosomes (superscaffolds). The anchored rate was 99.23%, and the N50 values of the contig and super-scaffold were 2.43 Mb and 127 Mb, respectively (**Table 1** and **Supplementary Table S6**), and each chromosome was ~ 120 Mb in length (**Table 1** and **Supplementary Table S7**). Subsequently, the Hi-C interaction heatmap showed that the interaction at the diagonal position was significantly higher than at other positions, demonstrating a better assembly effect (**Supplementary Figure S6**), which was also supported by the LAI evaluation results showing that most values were > 10 (**Supplementary Figure S7**). The BUSCO evaluation indicated that the contigs and scaffold assemblies covered 94.67% and 94.61% of the core conserved genes, respectively (**Table 1** and **Supplementary Table S8**).

**Table 1:** Assembly and annotation statistics of the *F. dibotrys* genome.

Feature	Value
Assembled genome size (Mb)	967.5
Chromosome number	8
Average length per chromosome (Mb)	121
N50 of contig (Mb)	2.43
N50 of super-scaffold (Mb)	127
GC content (%)	0.42
Complete gene percentage of contig (%)	94.67
Complete gene percentage of super-scaffold (%)	94.61
Number of protein-coding genes	40610

Repetitive sequences comprise most of the genome. Therefore, TRF, RepeatMasker, RepeatproteinMask, and *de novo* annotation data were integrated and redundancy was masked. The final results showed that repetitive sequences collectively occupy 0.58 Gb (62.47%) of the genome (**Supplementary Table S9**). Among these, LTRs were the most abundant, accounting for approximately 58%, followed by LINEs. The divergence rates of the four TE sequences indicated that most TE divergences were concentrated at ~ 30% (**Supplementary Figure S8**). Combining the *de novo* and homology-based methods ultimately yielded a gene set consisting of 40610 genes (**Supplementary Table S10**). Regarding genome annotation, 1523 (94.4%) of the 1614 Embryophyta single-copy orthologs from BUSCO were identified as complete genes in the *F. dibotrys* genome, indicating a high-quality genome assembly (**Supplementary Table S8**). Regarding ncRNAs, the annotation results indicated that rRNA had the highest proportion (0.066%), followed by snRNAs (0.010%). The lowest proportion was observed for tRNA (0.009%; **Supplementary Table S10**). Regarding gene functional annotation, after integrating the prediction results, 23037 genes were annotated using five databases, comprising 56.72% of the total (**Supplementary Figure S9**).

### 3.2 Evolutionary history and whole-genome duplication

**Figure 2:**
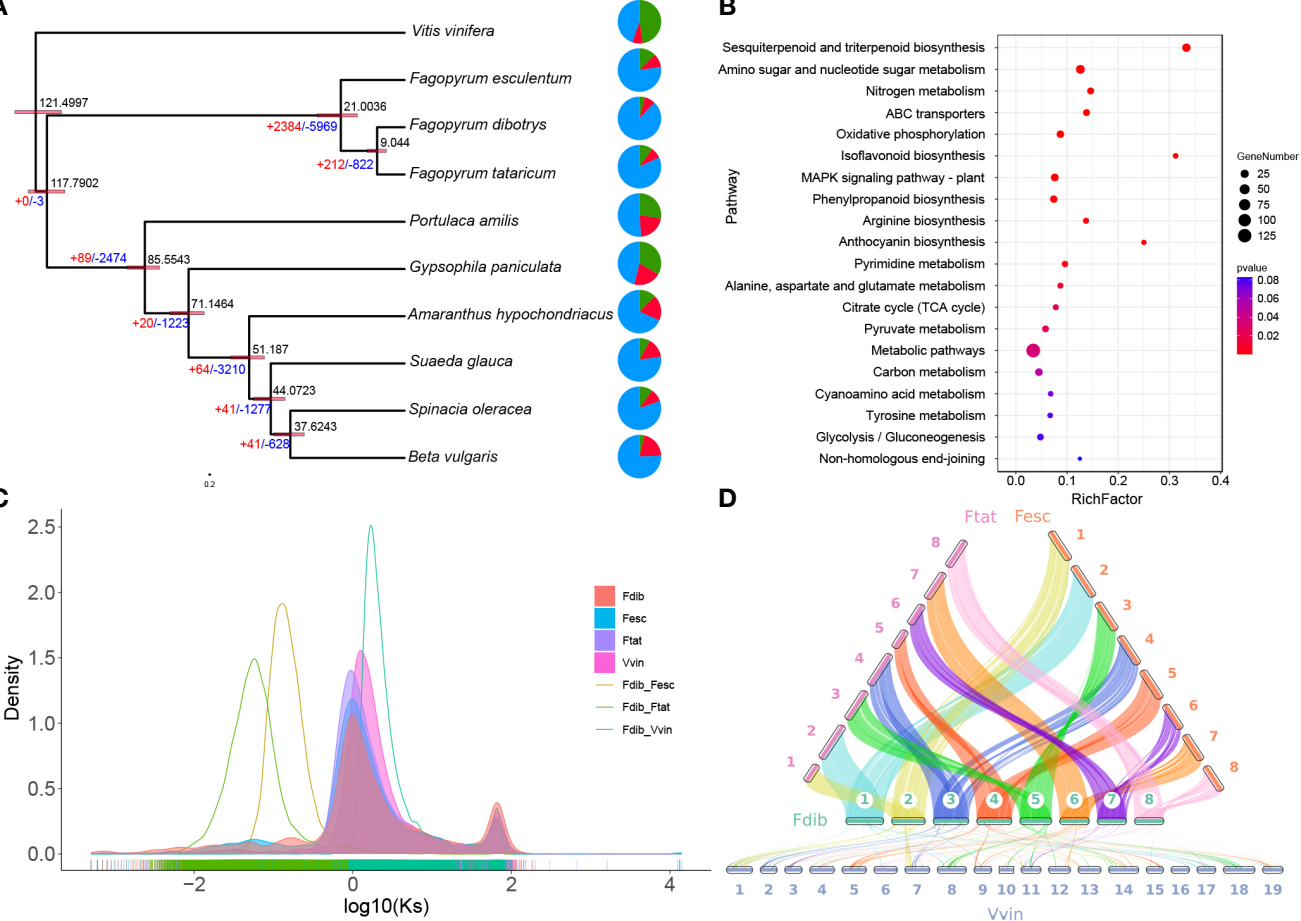
Evolution of the genome and enrichment of the expanded gene family. **(A)** Phylogenetic tree of Caryophyllales and the estimated divergence times are indicated at the internodes. The expanded and contracted gene families are represented in red and blue, respectively. **(B)** Significantly expanded gene families in *F. dibotrys* enriched using KEGG pathway analysis. **(C)** The frequency distribution of the *K_s_* values of homologous gene pairs. **(D)** The collinearity of *F. dibotrys*, *F*. *tataricum*, *F*. *esculentum*, and *V. vinifera*.

This study selected ten species, nine of *Caryophyllales*, and one of *V. vinifera* as an outgroup. These were clustered into 56424 gene families, including 328 single-copy genes, to infer the high-confidence phylogenies of these species. Using molecular clock analysis and calibrated with the TimeTree database, it was estimated that the genus *Fagopyrum* diverged from other genera in the *Caryophyllales* order 117.79 million years ago (Mya), *F. esculentum* divergence from the common ancestor of *F. dibotrys* and *F. tataricum* 21.00 Mya, and *F. dibotrys* diverged from *F. tataricum* 9.04 Mya (**Figure 2A**). The evolution of gene families plays a crucial role in the phenotypic variation in plants. Based on the analysis of the expansion and contraction of gene families in *F. dibotrys*, 1733 expanded gene families were detected, primarily enriched in sesquiterpenoid and triterpenoid biosynthesis, amino sugar and nucleotide sugar metabolism, and metabolic pathways (**Figure 2B**). Based on the phylogenetic tree, *F. tataricum, F. esculentum*, and *F. dibotrys* are closely related. Collinearity analysis revealed strong collinearity among *F. tataricum*, *F. esculentum* and *F. dibotrys* (**Figure 2D**).

WGD or polyploidy events, is common in plant genomes and contributes to variations in both size and content among plants (Soltis & Soltis, 2016). Considering the Log_10_ of the distribution of synonymous substitutions per synonymous site (*K_s_*) value was used to increase the distance between different *K_s_* peaks and better distinguish different WGD events (**Figure 2C** and **Supplementary Figure S10**). Unlike the *K_s_* peak in the *V. vinifera* genome, the *K_s_* values for paralogous genes in the collinear regions of *F. dibotrys*, *F. tataricum*, and *F. esculentum* appeared to show the same *K_s_* peak, indicating that they shared the same WGD event before the divergence of the *Fagopyrum* species and that no WGD event occurred in *Fagopyrum* after the divergence event. Although they have a close evolutionary relationship, they exhibit significant differences in genome size, *F. esculentum* 1,219.3 Mb, *F. tataricum* ~ 453.9 Mb (He et al., 2023), and *F. dibotrys* 967.5 M (**Table 2**). In *Fagopyrum*, genomes with large sizes also have a higher proportion of repetitive sequences *F. esculentum* ~ 72.9%, 888.9 Mb, *F. tataricum* ~ 52.4%, 237.8 Mb, *F. dibotrys* ~ 62.5%, 604.7 Mb (**Table 2**), and the gene number is similar (*F. esculentum* 38078, *F. dibotrys* 40610, *F. tataricum* 34366). Compare genome size and the repetitive sequences among these three species, the repetitive sequences (*F. esculentum* - *F. dibotrys* 284.2 Mb, *F. esculentum* - *F. tataricum* 651.1 Mb, *F. dibotrys* - *F. tataricum* 366.9 Mb) seems the main reason for the different genome size, consistent with previous report (He et al., 2023).

**Table 2:** Assembly and annotation of the *F. dibotrys*, *F. esculentum*, and *F. tataricum* genome.

Feature	*F. dibotrys*	*F. esculentum*	*F. tataricum*
Genome size (Mb)	967.5	1,219.3	453.9
N50 of contig (Mb)	2.43	2.4	27.9
N50 of super-scaffold (Mb)	127	154.3	56.7
GC content (%)	0.42	0.39	0.38
Repeat region of Genome (%)	62.5	72.9	52.4
Predicted Gene number	38,078	40,610	34,366

### 3.3 Evolution of MADS-box genes family and their chromosomal localizations

**Figure 3:**
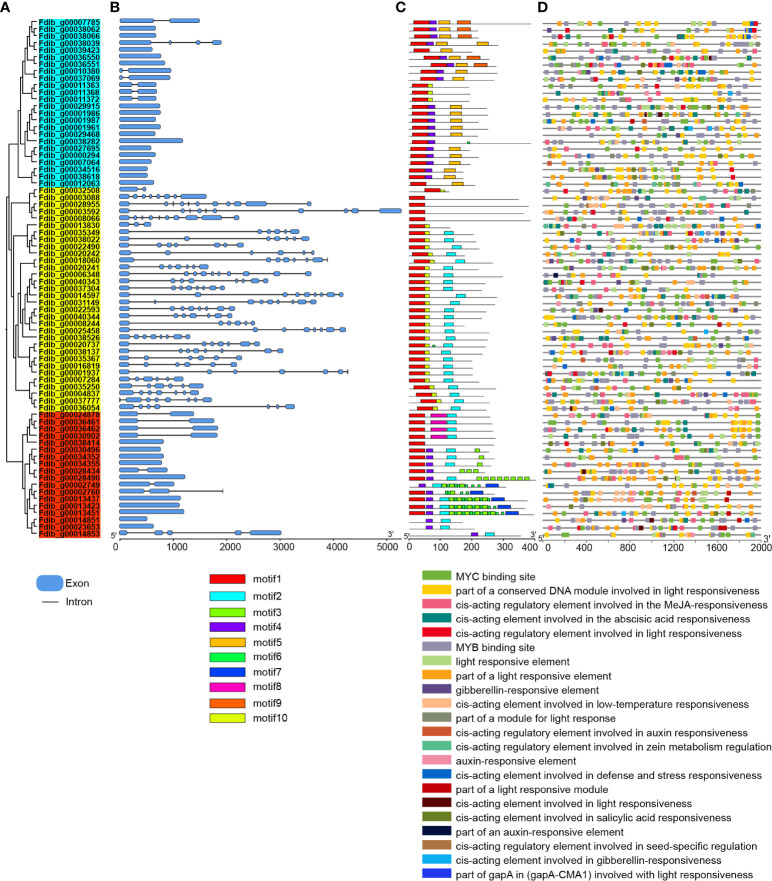
Phylogenetic tree, gene structures, conserved protein motifs and cis-elements analysis of MADS-box in *F*. *dibotrys*. **(A)**: Phylogenetic tree of MADS-box, light blue part represents Mα subgroup, red part represents Mγ subgroup, yellow part represents MIKC-type subfamily, **(B)**: gene structures of MADS-box, blue box represent exons, black line represent intron, **(C)**: conserved protein motifs MADS-box, different color represents different motif, d: cis-elements analysis of upstream 2000bp promoter sequence of MADS-box, different colors represent different elements.

*A. thaliana* MADS-box genes were used as query sequences. We identified 74 homologs in the *F. dibotrys* genome, in comparison to *F. tataricum* and *F. esculentum* within the same genus and 62 and 89 homologs, respectively (**Supplementary Table S11** and **Supplementary Figure S11**). Among these, *F. esculentum* (54 copies) had more MIKC-type MADS-box genes than *F. dibotrys* (32 copies) and *F. tataricum* (40 copies) (**Supplementary Figure S11**). Moreover, analysis of whole-genome duplication revealed that they underwent the same WGD event before the divergence, and no WGD event occurred after the divergence event. From this we inferred that the additional copies of MIKC-type MADS-box might result from gene family expansion and the obvious differences of MADS-box members in genus *Fagopyrum* might be related to aspects of plant development including flower and embryo development (Schilling et al., 2020). To further analyze the structure of the MADS-box in the *F. dibotrys* genome, intron-exon analysis revealed that the vast majority of M-type (light blue and red parts) MADS-box genes have one or two introns, and most of them lack introns. In contrast, the number of exons and introns was high in MIKC-type (yellow part) subfamily; the number of exons from 1–11 and the number of introns from to 1–10 (**Figure 3B**), and two subgroups (**Figure 3A**, Mα: light blue part and Mγ: yellow part) had similar gene structures. Additionally, the same subgroup contained relatively conserved and similar motif compositions, indicating that these genes shared the basic general characteristics of the MADS-box gene family. Furthermore, motif 4 is unique in M-type subfamily, motif 9 and motif 5 are unique in Mα subgroup but Mα subgroups lack of motif 2 (**Figure 3C**). Motif 3 and motif 7 only identified in Mγ subgroup, this result is consistent with the result of subfamily classification. Based on the 2000 bp up-steam sequence analysis of the MADS-box in *F. dibotrys* genome, these cis-acting elements mainly contain light-responsive elements, hormone-responsive elements, and MYB-binding sites (**Figure 3D**). In the *F. dibotrys* genome, the majority of these were type I genes distributed across every chromosome, spanning nearly all subfamilies, and the Mα subfamily constituted the majority with 24 copies and was present on almost all chromosomes. Moreover, type I genes exhibited a tandem distribution, particularly the Mγ subfamily on chromosome 6. The *F. dibotrys* genome contained genes corresponding to the ABCE model, including four A-class genes from the SQUA subfamily, potentially determining sepal identity; three B-class genes from the DEF subfamily that, in combination with the A-class genes, might control petal identity; two C-class genes from the AG subfamily that were likely involved in stamen identification along with the B-class genes; and six E-class genes from the AGL2 subfamily, which have specific roles as mediators of higher-order complex formation (**Supplementary Figure S12** and **Supplementary Figure S13**).

### 3.4 Mining genes involved in the biosynthesis of flavonoids

**Figure 4:**
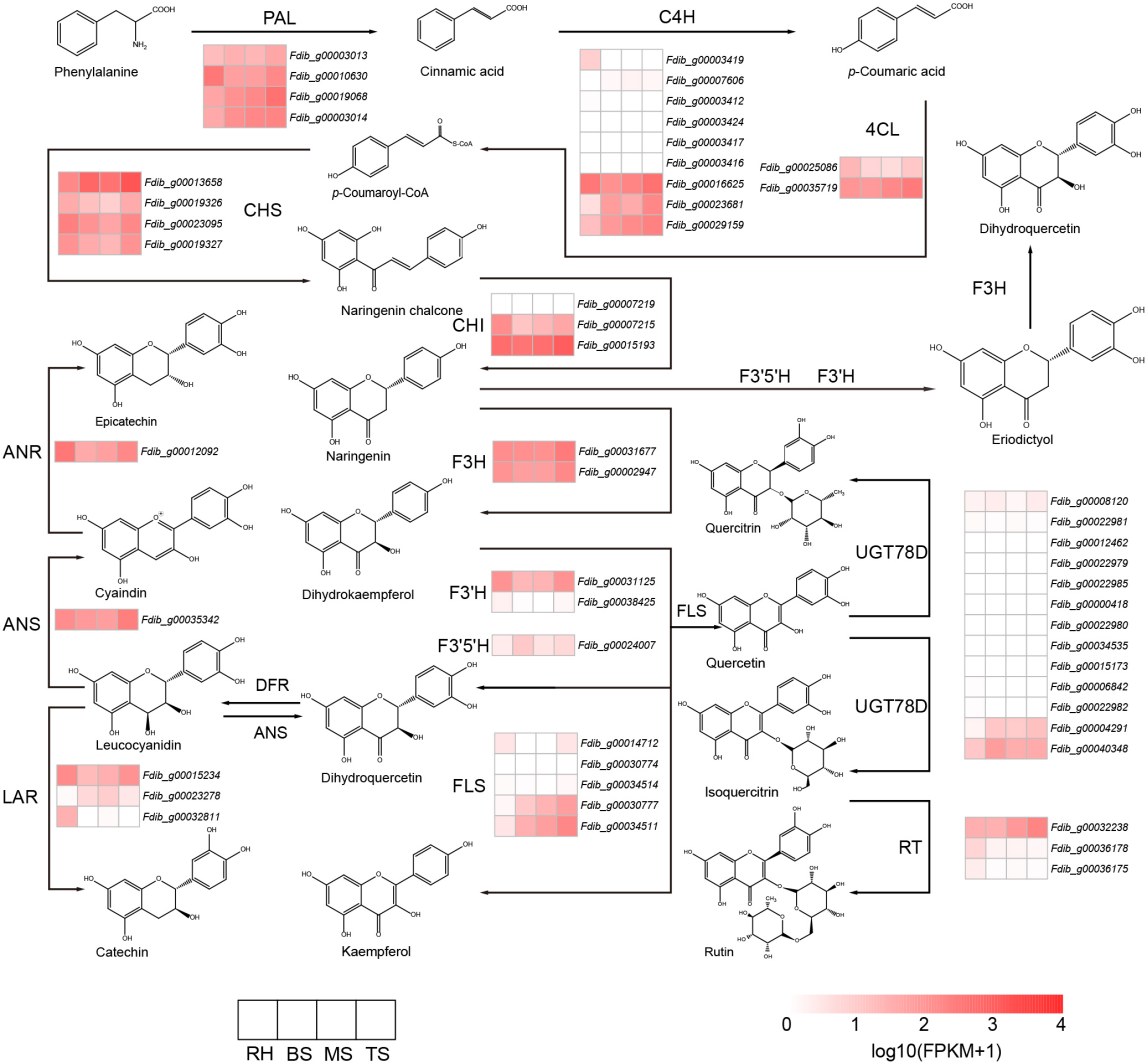
Flavonoid metabolic pathway and expression analysis of the genes involved. PAL: (phenylalanine ammonia-lyase, C4H: cinnamate 4-hydroxylase, 4CL: 4-coumarate–CoA ligase, CHS: chalcone synthase, CHI: chalcone isomerase, F3H: flavanone 3- hydroxylase, F3’H: flavonoid 3’-hydroxylase, F3’5’H: flavonoid 3’,5’-hydroxylase, FLS: flavonol synthase, ANR: anthocyanidin reductase, ANS: anthocyanin synthase, LAR: leucoanthocyanidin reductase, RT: flavonol 3-O-glucoside 6-O-rhamnosyltransferase, UGT78D: UGT78D1: flavonol-3-O-rhamnosyltransferase; UGT78D2: flavonol 3-O-glucosyltransferase.

*F. dibotrys* accumulates numerous critical bioactive compounds, such as flavonoids, phenolics, fagopyritols, triterpenoids, steroids, and fatty acids (Jing et al., 2016). In *F. dibotrys*, various flavonoid compounds are initially derived from phenylalanine as substrates, catalyzed by multiple enzymes. In the genome of *F. dibotrys*, 4 *PAL*s (phenylalanine ammonia-lyase) and 4 *CHS*s (chalcone synthase), 3 *CHIs*: (chalcone isomerase), 9 *C4H*s (CYP73A: cinnamate 4-hydroxylase), 5 *FLS*s (flavonol synthase), 13 *UGT78D*s (UGT78D1: flavonol-3-O-rhamnosyltransferase; UGT78D2: flavonol 3-O-glucosyltransferase), 3 *LARs* (leucoanthocyanidin reductase), 3 *RTs* (UGT79A6: flavonol 3-O-glucoside 6-O-rhamnosyltransferase), 2 *4CLs* (4-coumarate–CoA ligase), 2 *F3’Hs* (CYP75B1: flavonoid 3’-hydroxylase), 2 *F3Hs* (flavanone 3-hydroxylase), 1 *ANR* (anthocyanidin reductase), 1 *ANS* (anthocyanin synthase), and 1 *F3’5’H* (flavonoid 3’,5’-hydroxylase) were identified through homologous search (**Figure 4**).

From dihydrokaempferol to dihydroquercetin, flavonoid biosynthesis was catalyzed by F3’H and F3’5’H enzymes, which belong to the cytochrome P450 family. Based on the phylogenetic tree result of the *CYP* gene (**Supplementary Figure S14**), we identified 1 *F3’5’H* gene and 2 *F3’H* genes in the genome of *F. dibotrys*, *F. esculentum*, and *F. tataricum*; we identified 2 *F3’5’H* genes and 1 *F3’H* gene in *R. tanguticum* genome. However, no candidates for the *F3’5’H* and *F3’H* gene could be identified in the genome of *A. hypochondriacus* and *S. glauca*. In other *Caryophyllales* species, only one *F3’H* gene was identified, but no candidate for the *F3’5’H* gene was identified (**Supplementary Table S12**). The absence of *F3’5’H* and *F3’H* genes in certain species might impact flavonoid biosynthesis and diversity. We inferred that these species lacked these metabolic pathways during the speciation process. Additionally, the phylogenetic tree of *F3’H* indicated that the different number of *F3’H* genes was speculated to be produced by the expansion of the gene family (red branch) and this phenomenon only occurs in the genus *Fagopyrum* (**Supplementary Figure S16B**). UGTs convert quercetin to quercitrin and rutin. Based on the phylogenetic tree results of the *UGT* genes (**Supplementary Figure S15**), we identified 3 *RTs* genes in the *F. dibotrys*, *F. esculentum*, and *F. tataricum* genomes, 13 *UGT78D* genes in the *F. dibotrys* genome, 9 *UGT78D* genes in *F. tataricum* genome, and 12 *UGT78D* genes in *F. esculentum* genome. In the *R. tanguticum* genome, 2 *RT* genes and 4 *UGT78D* genes were identified (**Supplementary Table S12**), and the number of *UGT* genes was lower than that in the *Fagopyrum* species. Compared with other species of *Caryophyllales*, we also identified fewer *RT* and *UGT78D* genes in *Fagopyrum* species. The obvious differences in UGT78D members among various species might be related to the accumulation and diversity of flavonoid glycosides. *Fagopyrum* had more gene copies of the *UGT78D* gene and *RT* gene compared with the other species of *Caryophyllales*, especially the *UGT78D* gene. We identified a substantial expansion in the *UGT78D* gene family (**Supplementary Figure S17A** red branch and **Supplementary Table S12**), and gene family expansion also occurred in the *RT* gene family (**Supplementary Figure S17B** red branch), which appears to be common among the Polygonaceae family. We inferred that the significant expansion of the *UGT78D* gene family might benefit flavonoid accumulation. Additionally, by calculating the gene expression levels related to flavonoid biosynthesis in different tissues of *F. dibotrys*, we found that during the withering stage, *ANR* and *ANS* were continuously expressed, which is beneficial for the accumulation of epicatechins and catechin. In addition, the expression levels in the rhizome were higher than those in the stem, suggesting that the rhizome accumulated more epicatechins (**Figure 4**).

### 3.5 Genetic structure of the populations of *F. dibotrys*

Sichuan, Guizhou, and Yunnan Provinces are located in the southwestern region of China, and the Hengduan Mountains are predominant in this area. This region benefits from its distinctive natural conditions, fostering rich flora and fauna resources, making it a crucial place for wild medicinal herbs in China; this region is also one of the main producing areas of *F. dibotrys*. After quality control, the average Q20 and Q30 values for the re-sequenced data from different sources were 97% and 89%, respectively, indicating that the data were sufficient for mapping to the reference genome (**Supplementary Table S13**). Except for the 6–3A dataset, the average alignment rate was 98% and the average coverage rate was 85% (**Supplementary Table S13**). A total of 609,024,689 SNPs and 62,964,288 indels were identified (**Supplementary Table S14**) and were primarily concentrated in the intergenic regions (**Supplementary Table S15**).

**Figure 5:**
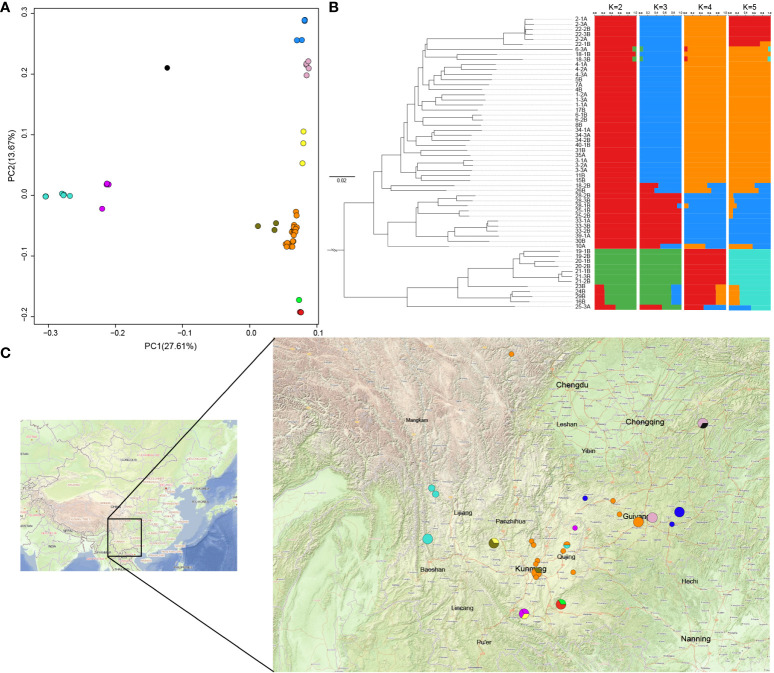
Genetic structure of the resequenced population. **(A)** PCA results of the resequenced individuals. Combining the results of population structure analysis, different colors represent the distinct subpopulations; Orange, blue, light blue, and red represent different homozygous subpopulations, while other colors represent heterozygous subpopulations, consistent with **Supplementary Table S1**. **(B)** Phylogenetic tree and population genetic structure of resequenced individuals. **(C)** Sampling site annotation, different-sized circles represent various sampling numbers (1–3), and different colors represent the different subpopulations.

Individual phylogenetic trees, PCA, and population structure analyses were combined based on the resequencing data. The population structures of the wild and cultivated varieties of *F. dibotrys* were initially investigated to determine the genetic backgrounds of the re-sequenced individuals. In conjunction with the PCA results, the re-sequenced individuals were initially classified into four homozygous subpopulations and six pairwise hybridization subpopulations. Further integration with the phylogenetic tree constructed based on the re-sequenced individuals suggested that the red subpopulation evolved from the orange subpopulation, further dividing it into three distinct homozygous subpopulations (**Figures 5A, B**, **Supplementary Figure S18, S19**). Geographically, a light-blue subpopulation was found in the southwestern part of Yunnan Province at the highest altitude, an orange subpopulation in the central part of Yunnan Province, and a blue subpopulation in the lower-altitude plains of Guizhou (**Figure 5** and **Supplementary Table S1**). The light blue subpopulation was located in the highest-altitude regions and differentiated first, followed by the orange subpopulation, with the blue subpopulation being the last to differentiate and was located in the lowest-altitude areas. Therefore, *F. dibotrys* is speculated to have originated from the high-altitude Tibetan Plateau. The cultivated varieties were bred using orange and red subpopulations from the central part of Yunnan Province as parental sources.

## 4 Discussion

*F. dibotrys*, *F. tataricum*, and *F. esculentum* belong to genus *Fagopyrum*, family *Polygonaceae*, and order *Caryophyllales*. *Fagopyrum* presently comprises 15 species (Jing et al., 2016). In contrast to *F. tataricum* and *F. esculentum*, which are staple food crops, *F. dibotrys* is a traditional Chinese medicinal herb with high therapeutic value. *F. dibotrys* was first recorded in “Bencaoshiyi”. In traditional Chinese medicine, the rhizome of *F. dibotrys* is used to treat lung abscesses, dysentery, rheumatism, throat inflammation, and tumefaction (Chen & Li, 2016) and its extraction of rhizome can condensed Tannin compound, which is used to prepare a medicine “WeiMaiNing” (Ke et al., 2021). As research progresses, the medicinal value of *F. dibotrys* has been discovered and received rising attention. The overexploitation of wild germplasm resources can deplete environmental resources and reduce species diversity. Wild genetic resources cannot meet the growing demands and require exploration for industrial cultivation.

In the present study, we assembled and annotated the genome of *F. dibotrys*, which had a total length of 967.5 Mb, and the genome sequence of *F. dibotrys* provided a valuable genomic resource for *Fagopyrum*. Phylogenetic analysis revealed that *Fagopyrum* underwent a single WGD event before species divergence. Additionally, they have a one-to-one syntenic relationship at the whole-chromosome level. When combined with previous research, the recent large-scale LTR bursts contributed to most of the genome size of *F. esculentum* (He et al., 2023). Subsequently, we inferred that the amplification of the repetitive sequences was the primary factor driving the variation in genome size among genus *Fagopyrum*. In addition, MADS-box genes are prime candidates; they are involved in virtually all aspects of plant development, and especially have an important impact on flower development (Schilling et al., 2020). We identified 74 MADS-box genes in the genome of *F. dibotrys* and found that these genes corresponded to the ABCE model of flower structure formation. Genome mining can considerably enhance the exploration of biosynthetic pathways for natural products. Flavonoids are one of the most important accumulations in the genus *Fagopyrum*, especially six major: quercetin, rutin, orientin, homoorientin, vitexin, and isovitexin (Joshi et al., 2020). While comparing gene copies number involved in flavonoid biosynthesis among different genera within *Caryophyllales*, we observed *Fagopyrum* possessed large number of copies of the *UGT78D*, *RT*, and *F3’H* genes compared with other species within *Caryophyllales*, particularly the abundance of copies within the *UGT78D* gene family relative to other genera. From this observation, we inferred that the *UGT78D* might have undergone expansion. Moreover, considering its role in catalyzing the biosynthesis of rutin and quercitrin, the expansion of the *UGT78D* family was crucial for the increased content of rutin and quercitrin in genus *Fagopyrum*. Additionally, we collected 46 wild samples and 12 cultivated varieties from Sichuan, Yunan, Guizhou, and Chongqing provinces, mainly located in the southwestern region of China, which is a critical area for producing Chinese medicinal herbs. By integrating the results of the population structure, PCA, and phylogenetic tree analyses, individuals from different regions were classified into four homozygous subpopulations based on topography, combining the evolutionary relationships that were further grouped into three homozygous subpopulations. Distributed across three distinct topographical areas, the light blue subpopulation was distributed at the highest altitude, followed by the orange subpopulation, whereas the blue subpopulation was distributed at the lowest altitude (**Figure 5** and **Supplementary Table S1**). Additionally, the cultivated varieties were based on the genetic background of the orange subpopulation and its evolved red subpopulation in central Yunnan Province. In a previous study, the southwestern region of China was thought to be the origin of *F. esculentum* and *F. tataricum* (Hunt et al., 2018), and *F. dibotrys* to be widely distributed in China, India, Thailand, and Vietnam (Zhang et al., 2021); however, phylogenetic trees indicated that they were wild relatives. Combining the resequencing results of our individuals, the light blue subpopulation of *F. dibotrys* situated at the high altitude of the Tibetan Plateau region was the first to complete differentiation, suggesting that *F. dibotrys* also originated in western China, and the Tibetan Plateau region appeared to be the more accurate region of origin for the *F. dibotrys* species.

Buckwheat in high-altitude regions seems to accumulate more flavonoids than that in low-altitude regions (Cheng et al., 2019; He et al., 2022). In addition, flavonoids protect plants against the harmful effects of UV radiation (Shen et al., 2022), considering that the light-blue subpopulation in high-altitude regions receives more intense UV radiation. Therefore, we speculate that the light-blue subpopulation would likely have the highest accumulation of flavonoids. Additionally, the cultivation base of this study was located in Kunming City, and collection of germplasm resources from higher altitudes indicated the need to overcome additional challenges. The cultivated varieties were based only on the genetic backgrounds of the orange and red subpopulations, indicating that the wild genetic resources used were limited to the vicinity of the cultivation base for an extended period. This restricted the genetic diversity of the populations that were bred, as the genetic advantages of different subpopulations could not be harnessed completely. Based on these theories, it is necessary to ascertain the content of relevant compounds in the light-blue subpopulation, and if they meet these requirements, further domestication can be considered. Additionally, the collection of wild genetic resources should be expanded, extending beyond the vicinity of Yunnan Province, with particular emphasis on the Tibetan region. The quality of medicinal herbs can be improved by harnessing the advantages of different subpopulations to ultimately serve human health needs. Therefore, it is necessary to expand the collection range of germplasm resources in the future, increase the genetic diversity of the population, enhance the quality of *F. dibotrys*, and reduce the overexploitation of wild resources.

## Data availability statement

The data presented in the study are deposited in the figshare repository, accession number: (https://doi.org/10.6084/m9.figshare.22240414.v1).

## Author contributions

S-HZ: Data curation, Formal analysis, Methodology, Software, Visualization, Writing – original draft, Writing – review & editing. Y-CD: Data curation, Formal analysis, Software, Visualization, Writing – original draft, Writing – review & editing. JD: Conceptualization, Data curation, Funding acquisition, Project administration, Writing – original draft, Writing – review & editing. J-TL: Conceptualization, Formal analysis, Resources, Validation, Writing – original draft, Writing – review & editing. SZ: Conceptualization, Formal analysis, Validation, Writing – original draft, Writing – review & editing. M-JL: Conceptualization, Software, Supervision, Validation, Writing – original draft, Writing – review & editing. H-CL: Formal analysis, Investigation, Resources, Supervision, Writing – original draft, Writing – review & editing. YZ: Conceptualization, Data curation, Formal analysis, Funding acquisition, Investigation, Methodology, Project administration, Resources, Software, Supervision, Validation, Visualization, Writing – original draft, Writing – review & editing. J-YW: Conceptualization, Investigation, Project administration, Supervision, Writing – original draft, Writing – review & editing.
