# Supplementary material for: Genomics and resequencing of Fagopyrum dibotrys from different geographic regions reveals species evolution and genetic diversity
Source: Front Plant Sci. 2024 Jun 11;15:1380157. doi: 10.3389/fpls.2024.1380157 (PMC11196786; doi:10.3389/fpls.2024.1380157)
Supplement: Supplementary file 1 [file DataSheet_1.docx]

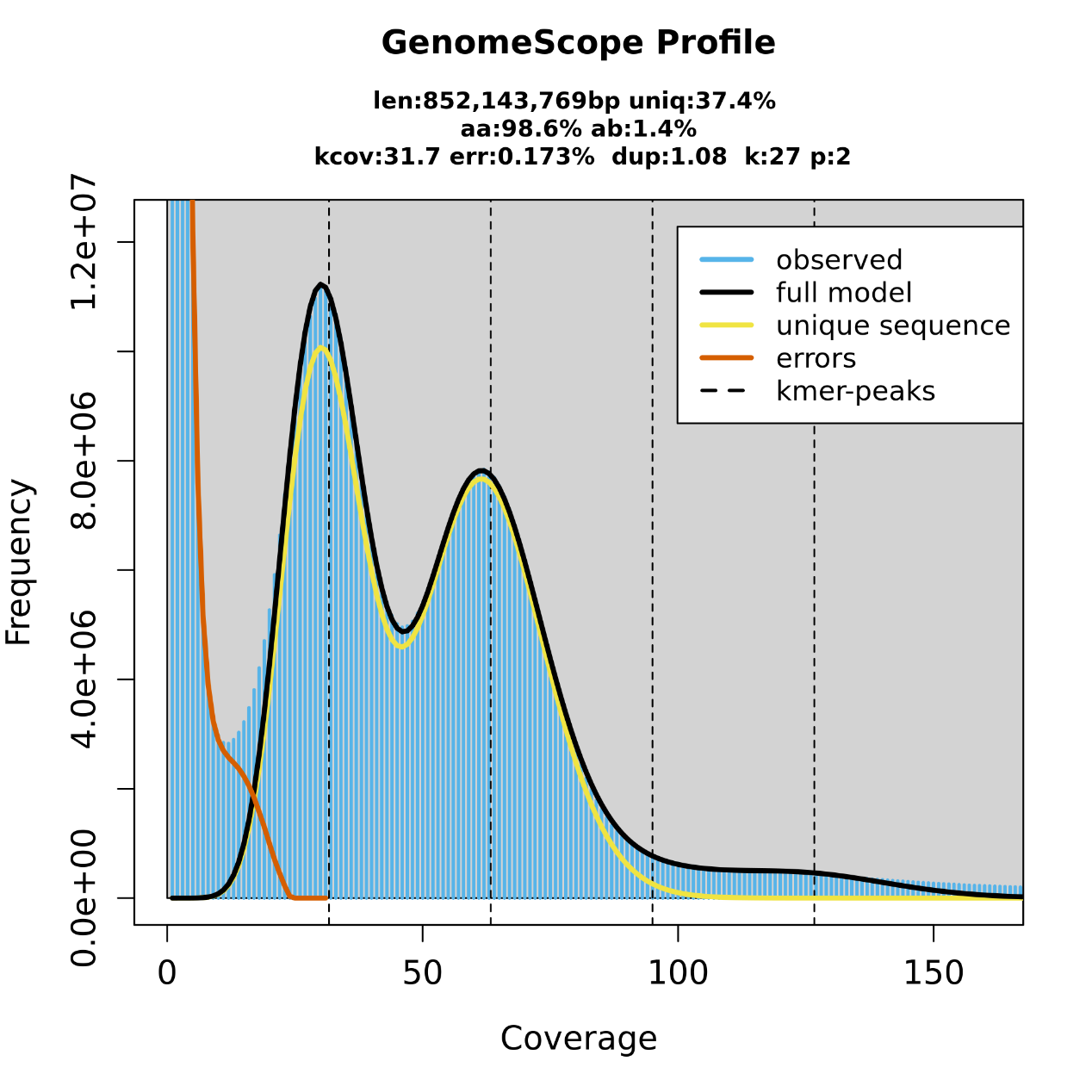


Figure S1 The frequency of 27 *k*-mer.


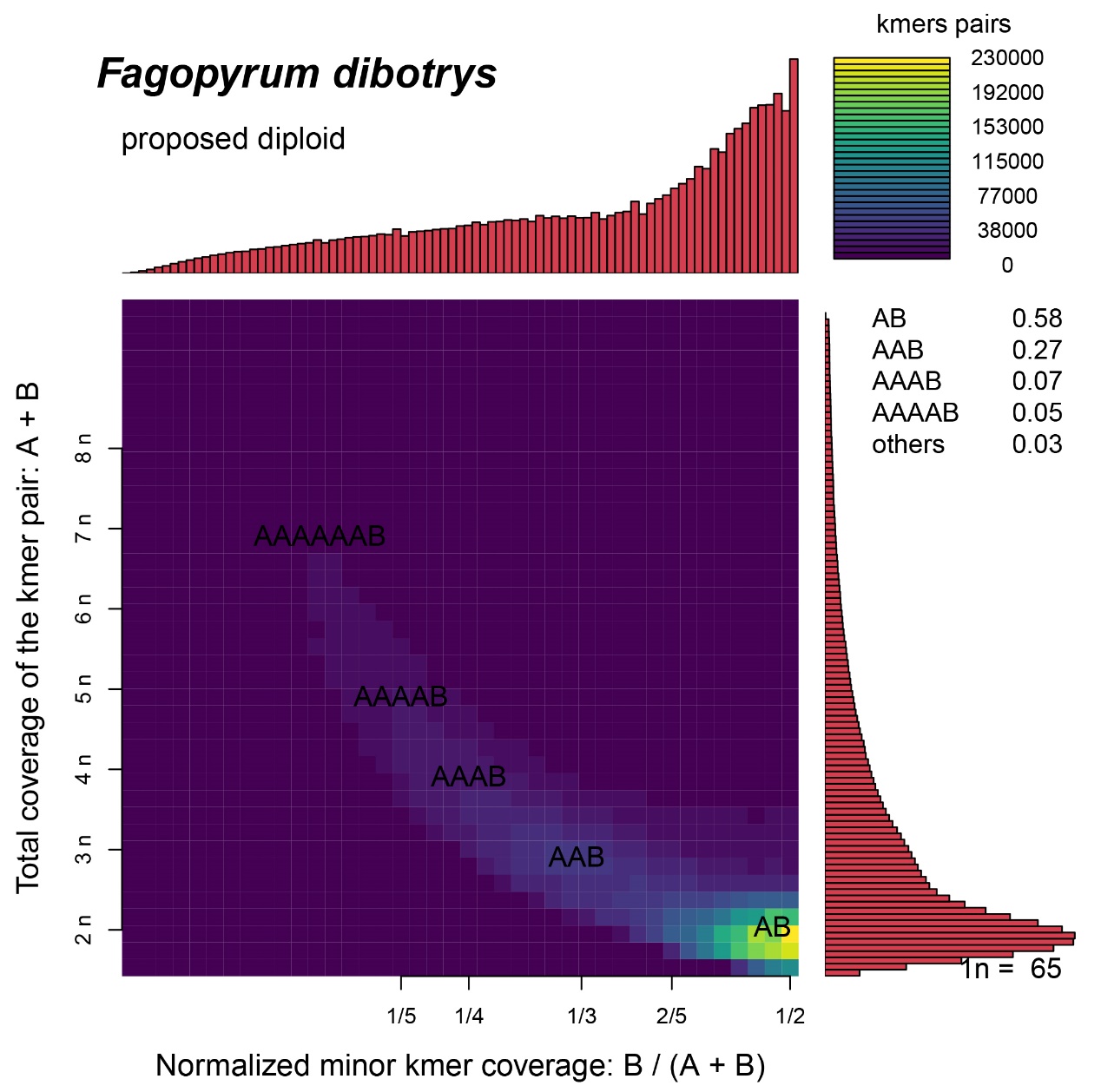


Figure S2 The result of Smudgeplot


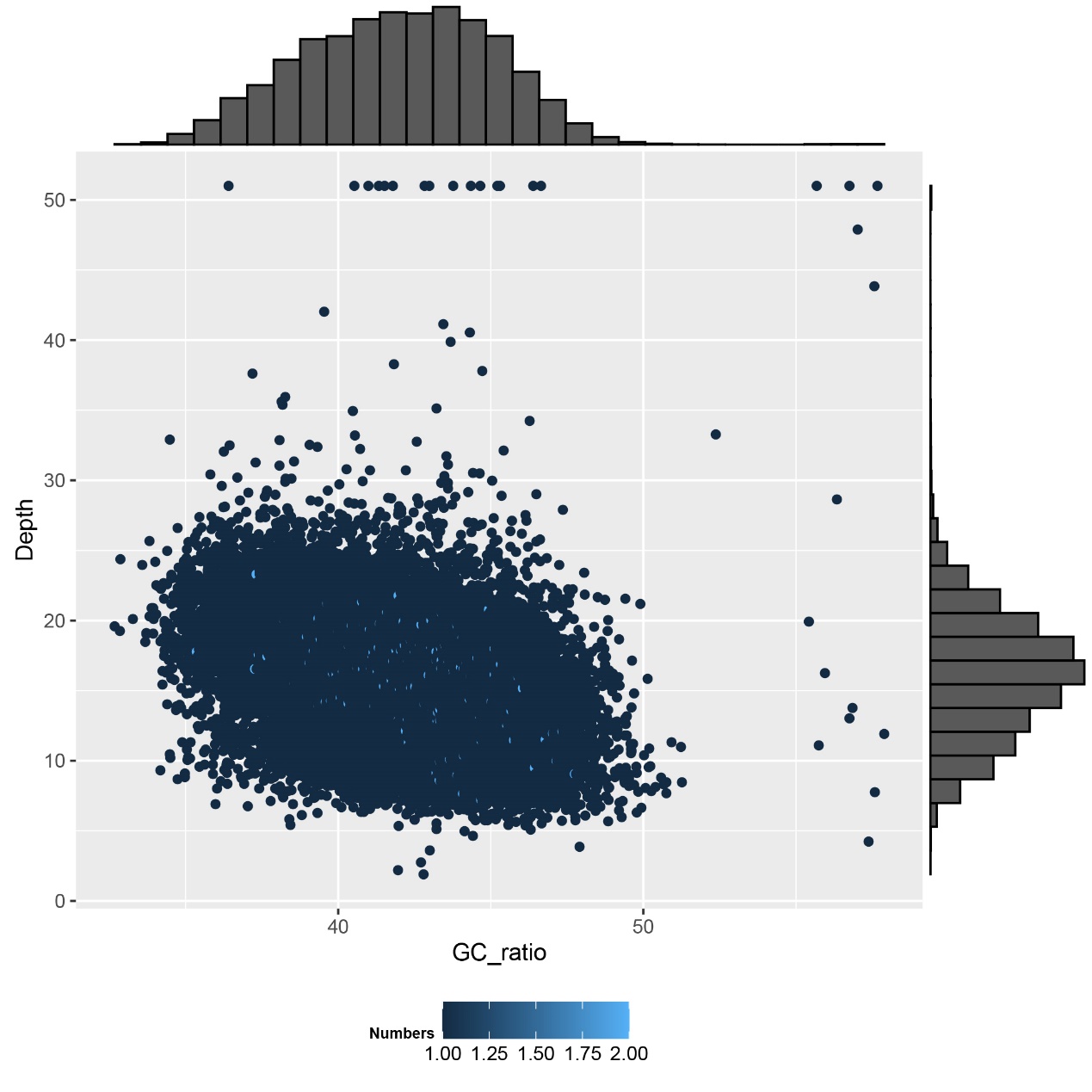


Figure S3 The GC ratio and depth result


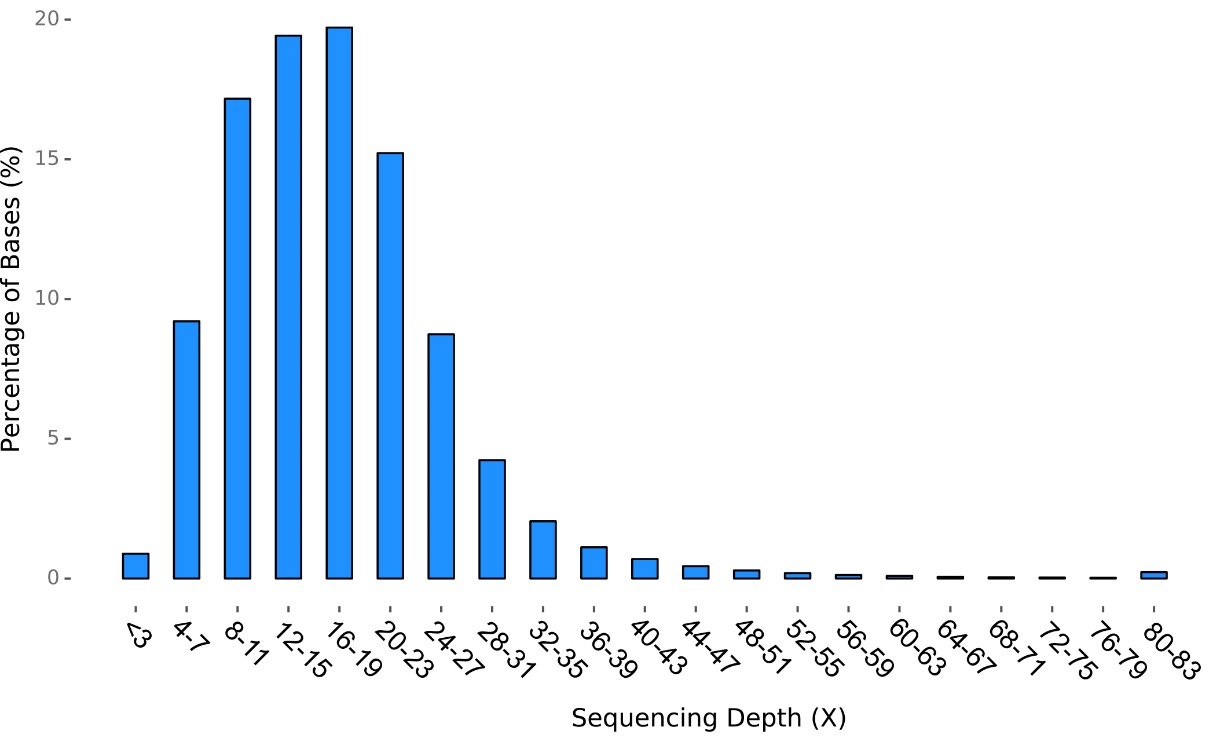


Figure S4 The Sequencing depth of *F. dibotrys.* The X-axis represents the coverage or depth of sequencing, indicating how many times each base in the genome has been sequenced. The Y-axis represents the proportion of total bases in the genome covered by the sequencing.


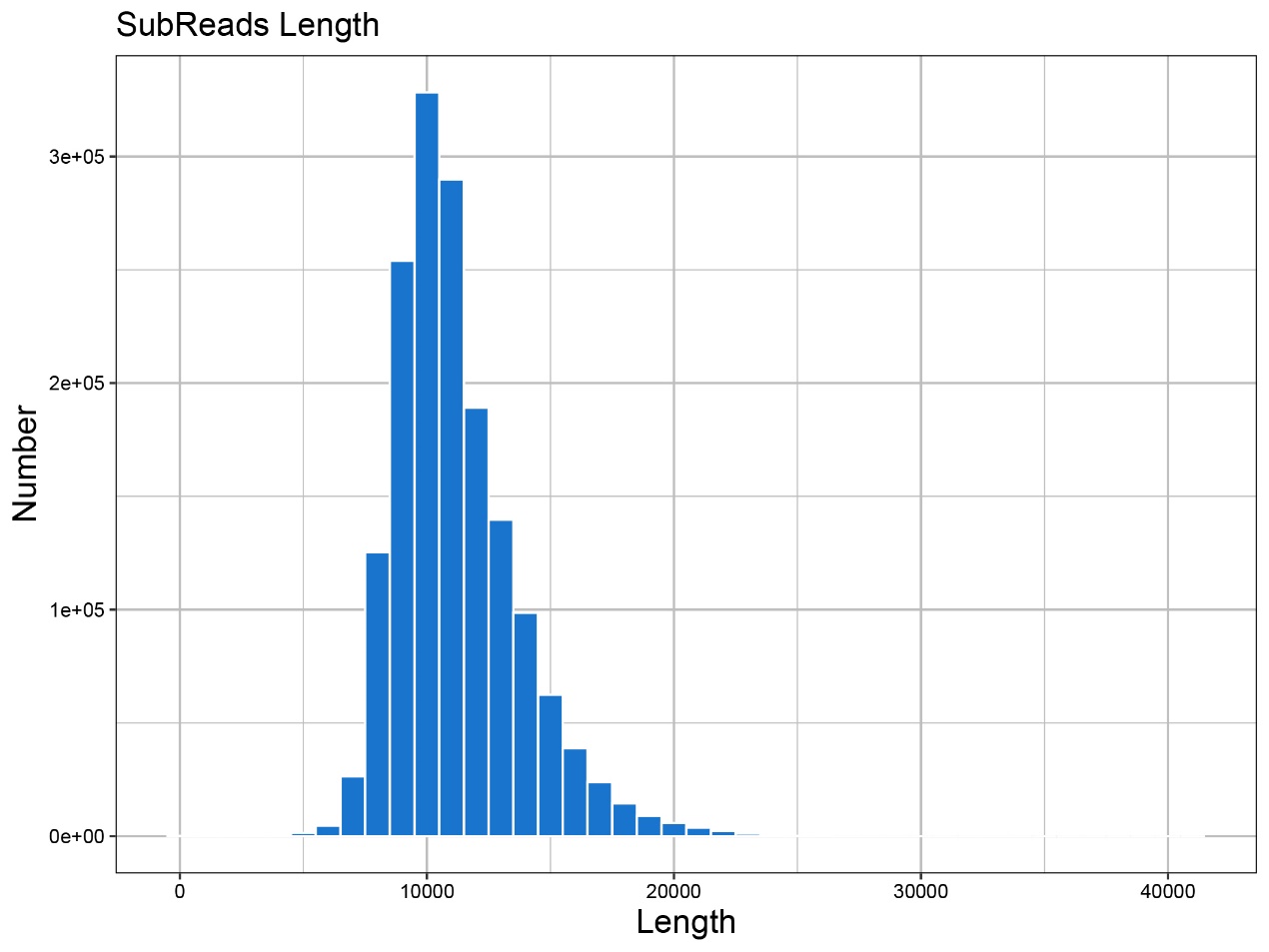


Figure S5 The single depth length number of the sequence. The X-axis represents the range of subread lengths obtained from the sequencing data (bp). The Y-axis indicates the count subreads corresponding to each specific length.


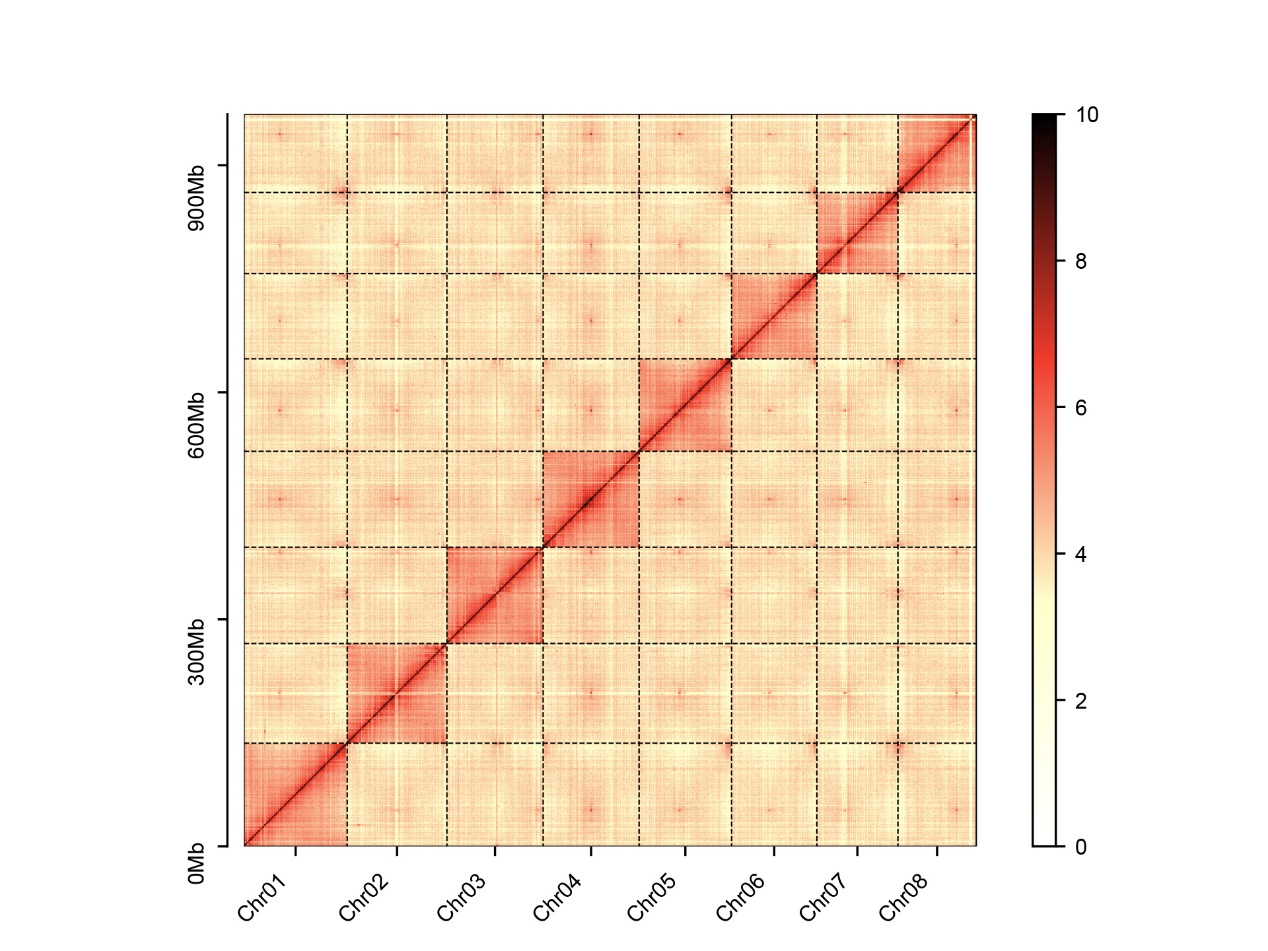


Figure S6 Genome-wide Hi-C map of *F. dibotrys.*


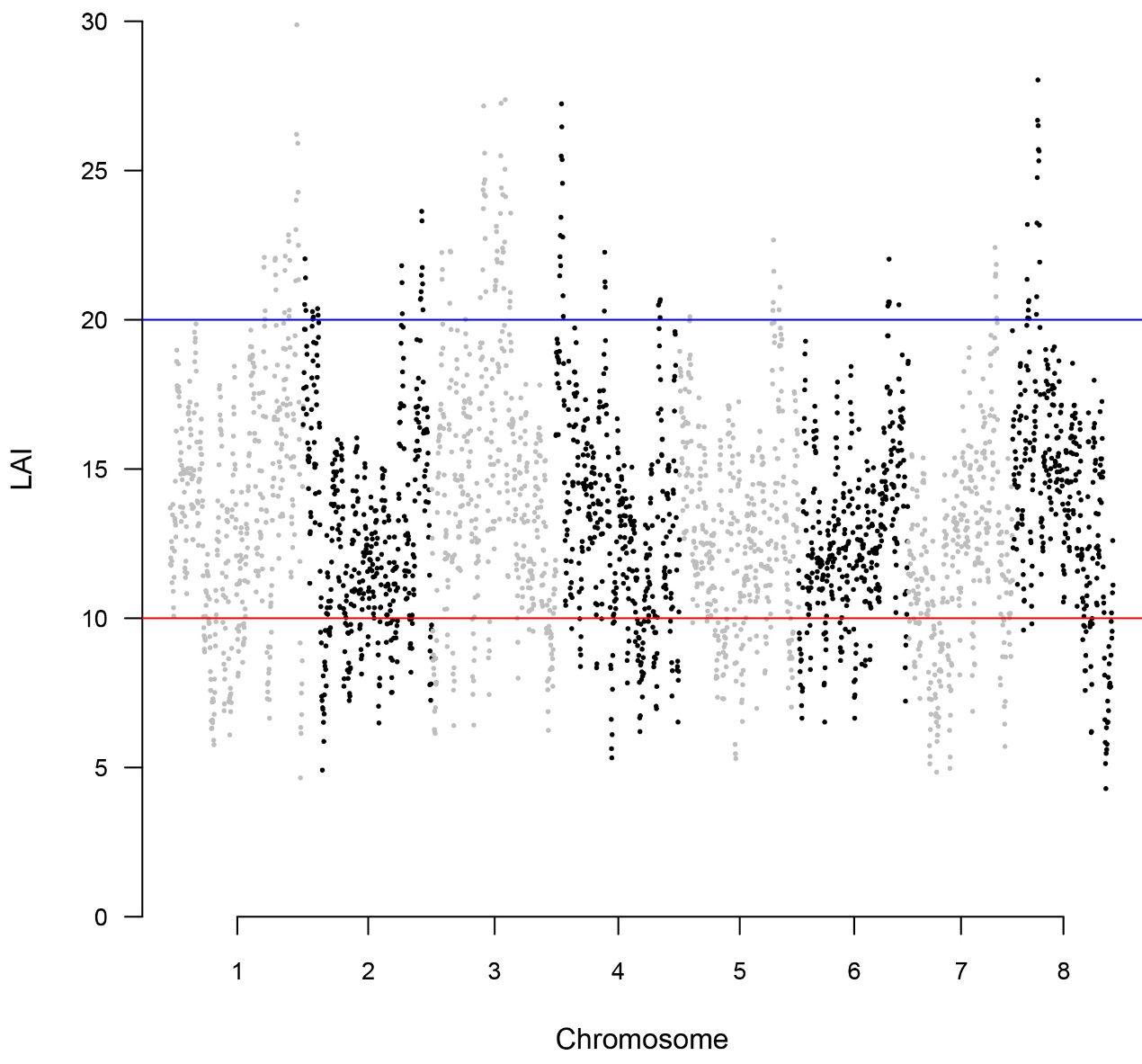


Figure S7 The LAI result of *F. dibotrys* genome. The x-axis represents different chromosomes.


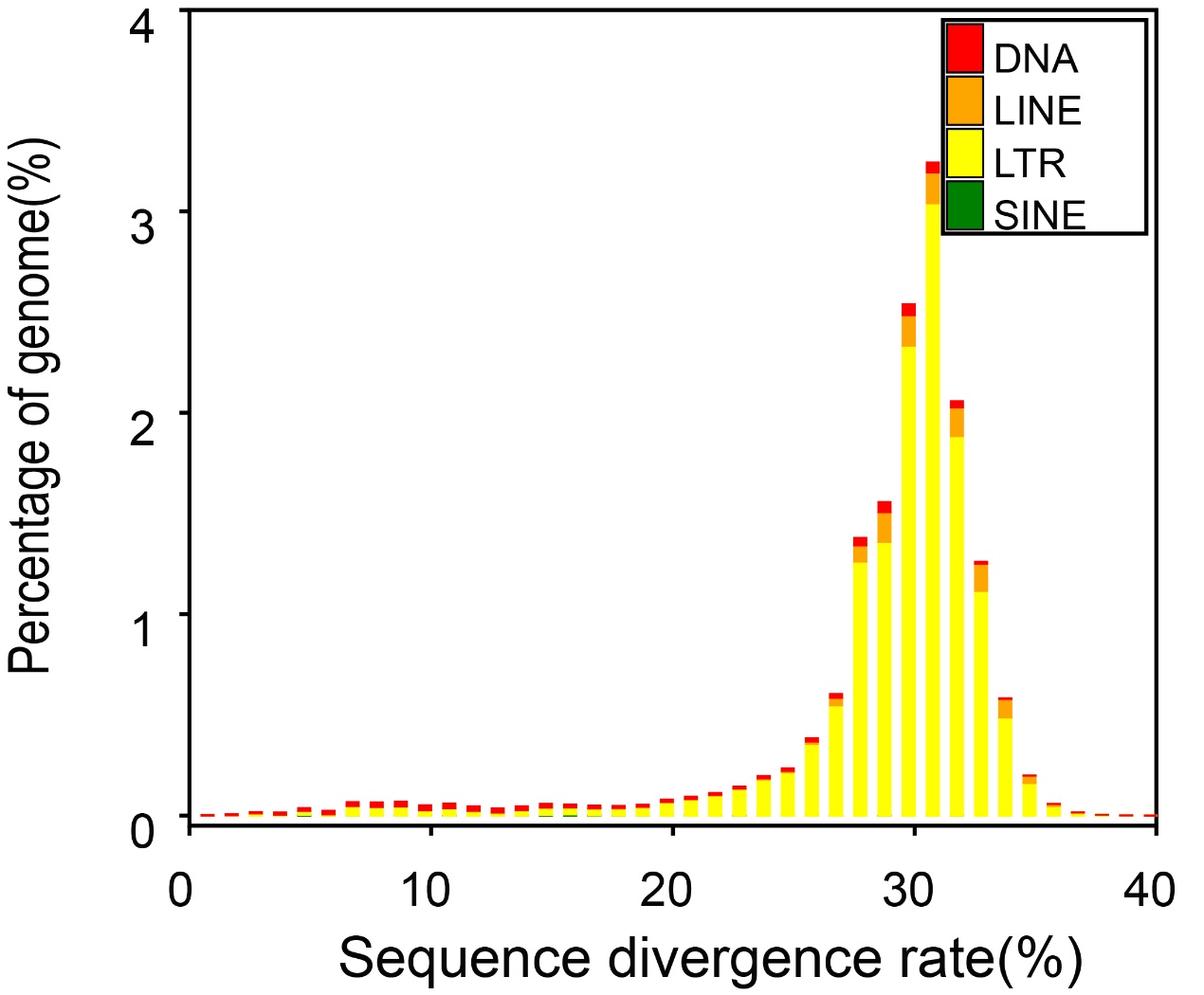


Figure S8 The sequence divergence rate of the genome. Different colors represent different transposon types. The X-axis represents the proportion of transposons, and the y-axis represents the sequence divergence rate.


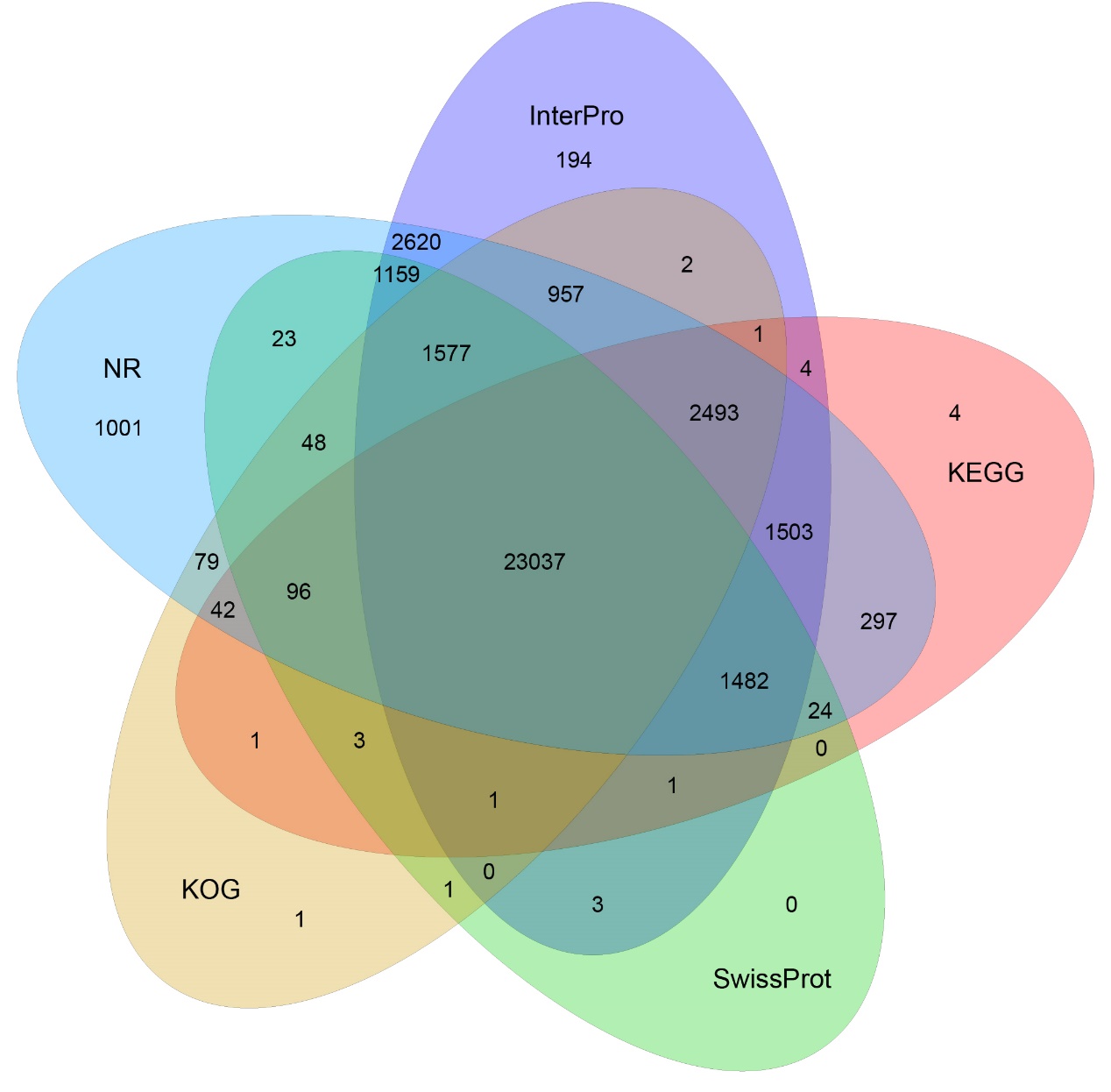


Figure S9 The functional gene annotation of *F. dibotrys* based on 5 databases. Different colors represented different databases.


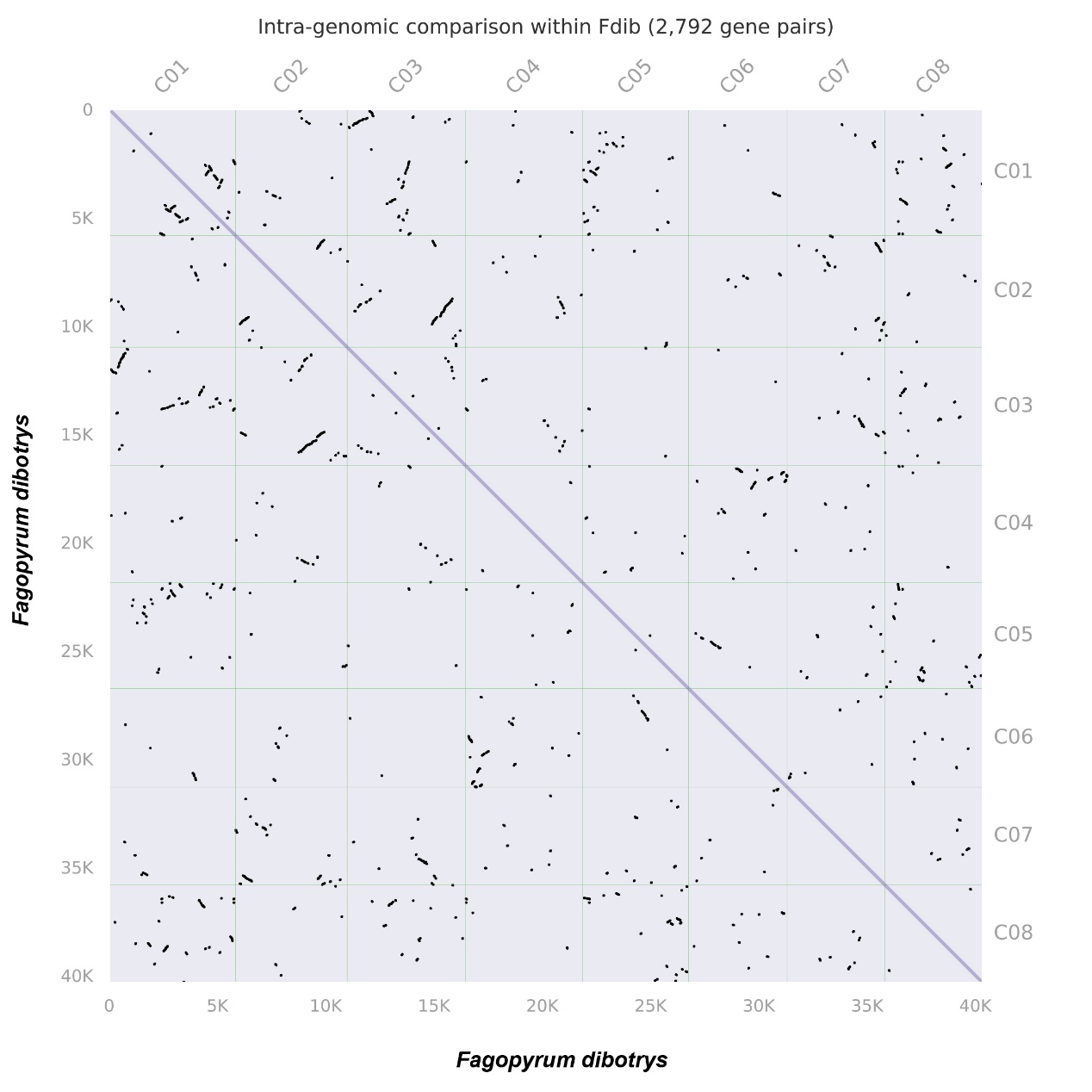


Figure S10 Dot plot of paralogous blocks in *F. dibotrys* shows the potential whole genome duplication event.


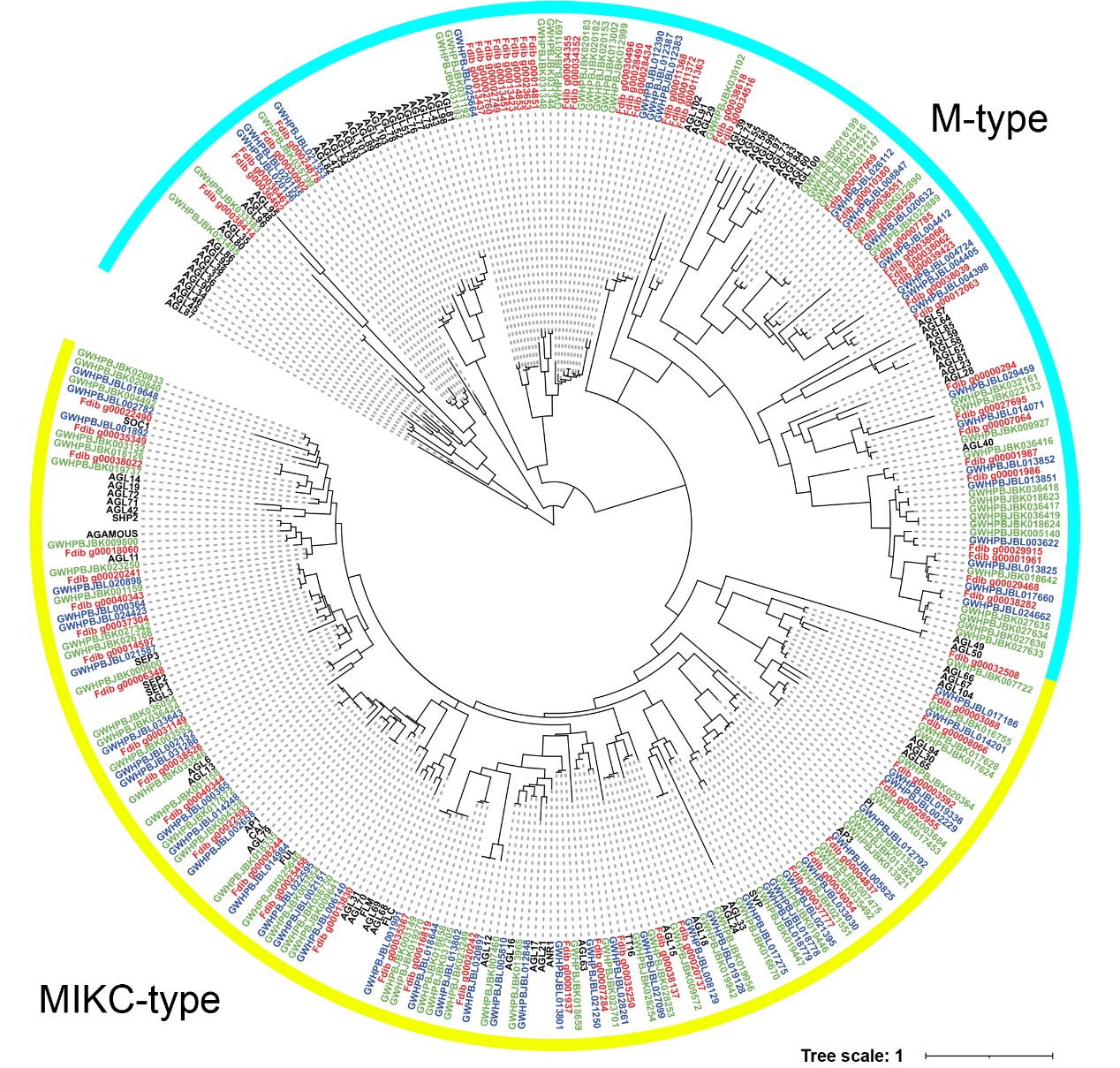


Fig S11 Phylogenetic tree of the MADS-box gene in *F. dibotrys*, *F. esculentum*, *F. tataricum*, and *A. thaliana*, red represents *F. dibotrys*, green represents *F. esculentum*, blue represents *F. tataricum*, black represents *A. thaliana*, different arcs represent different subfamily of MADS-box.


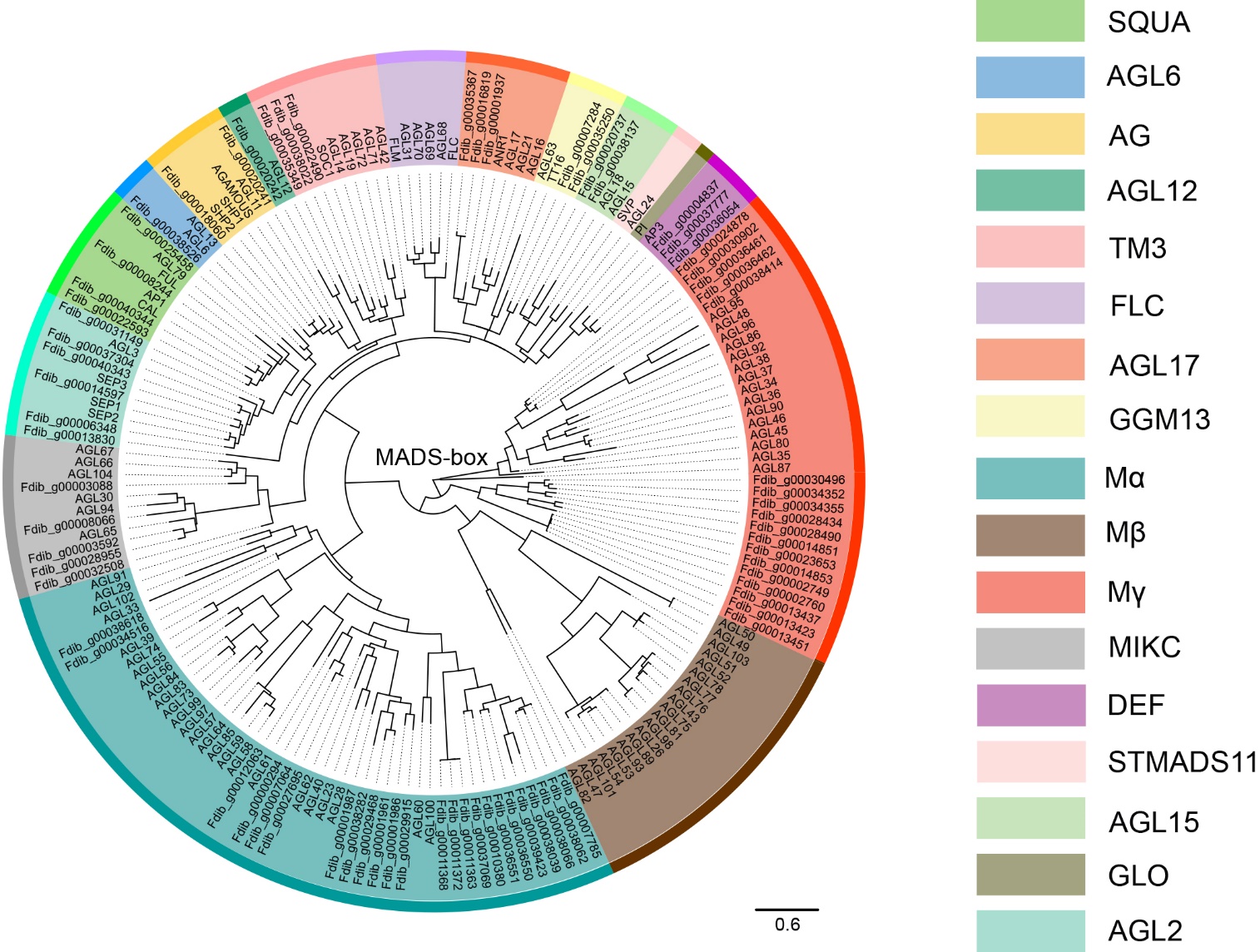


Figure S12 The phylogenetic tree of MADS-box genes. Distinct subfamilies differentiated by different colors.


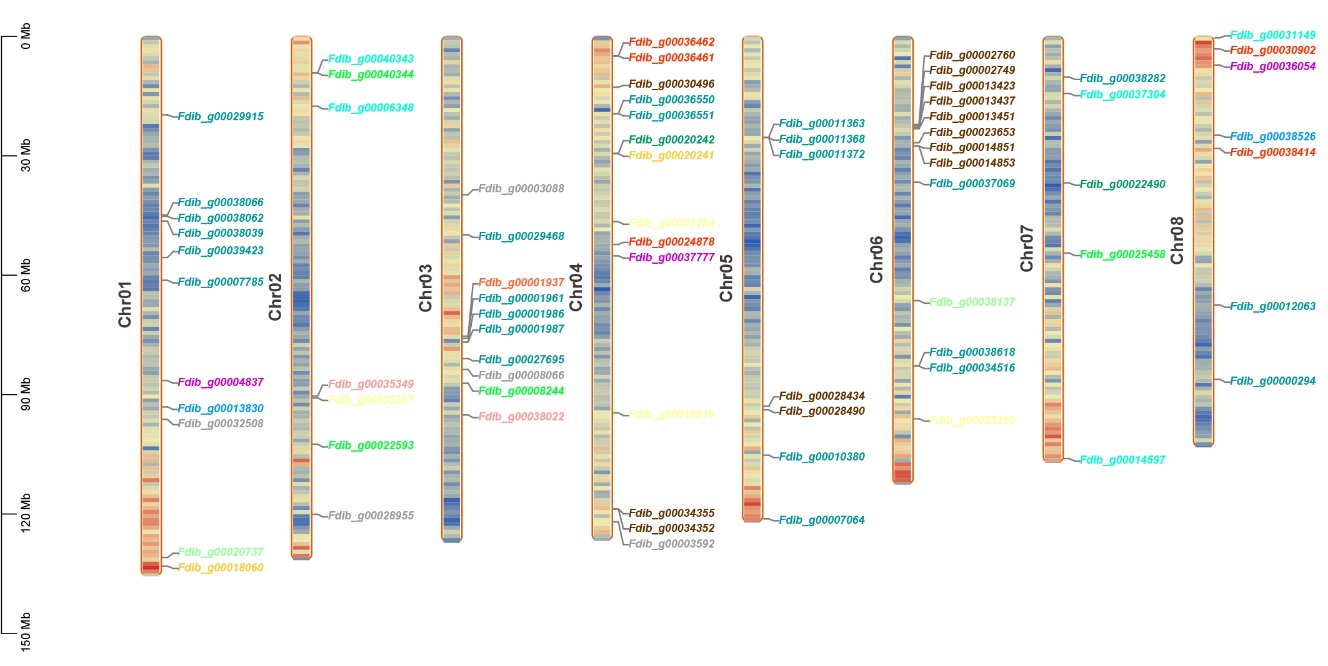


Figure S13 The localization of MADS-box genes on chromosomes, different colors represent distinct subfamilies.


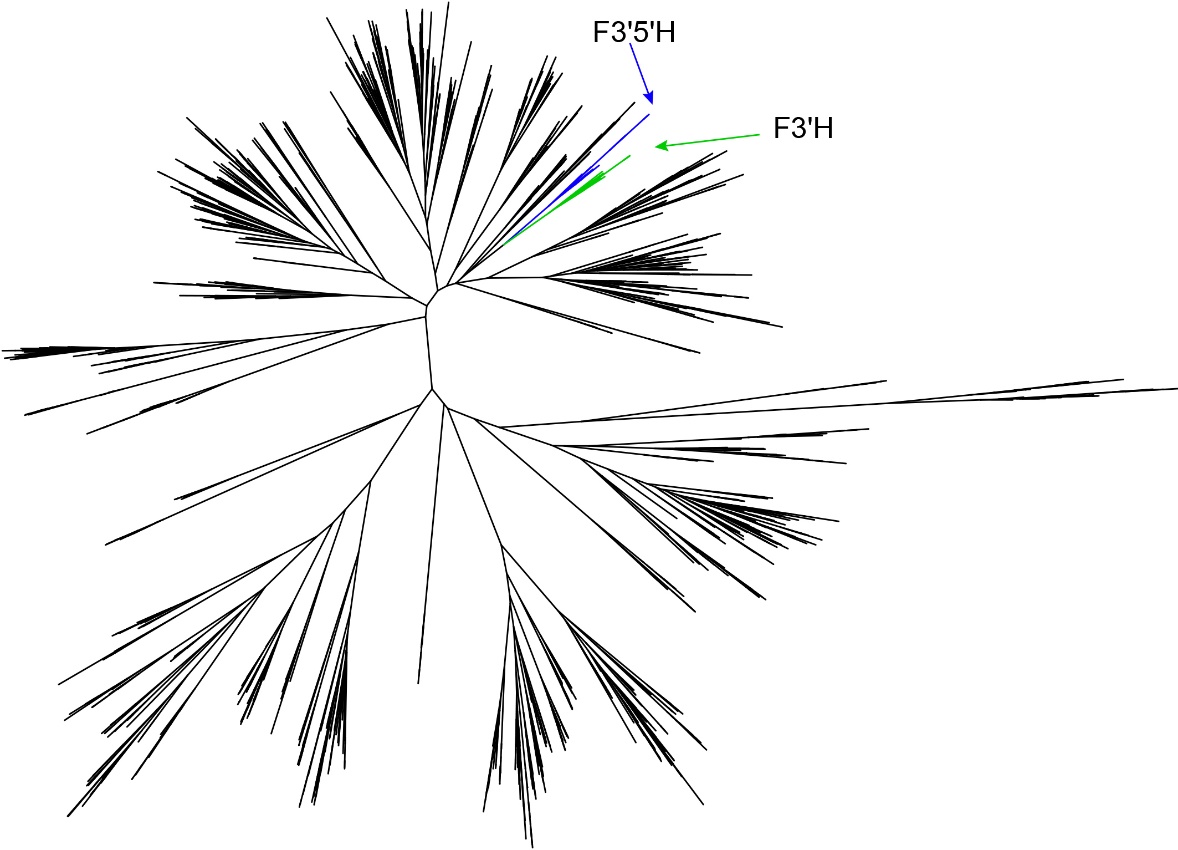


Fig S14 *CYP* gene phylogenetic tree. Blue represents the *F3'5'H* gene branch, and green represents the *F3'H* gene branch.


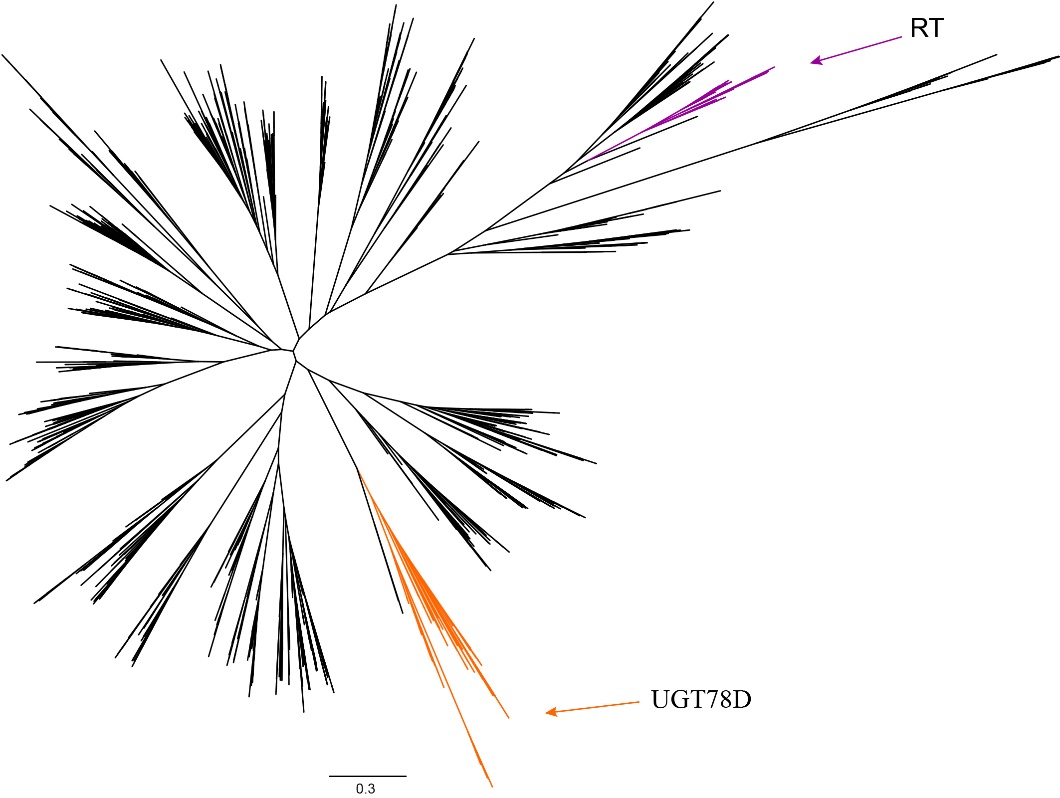


Fig S15 *UGT* gene phylogenetic tree. Purple represents the *RT* gene branch, and orange represents the *UGT78D* gene branch.


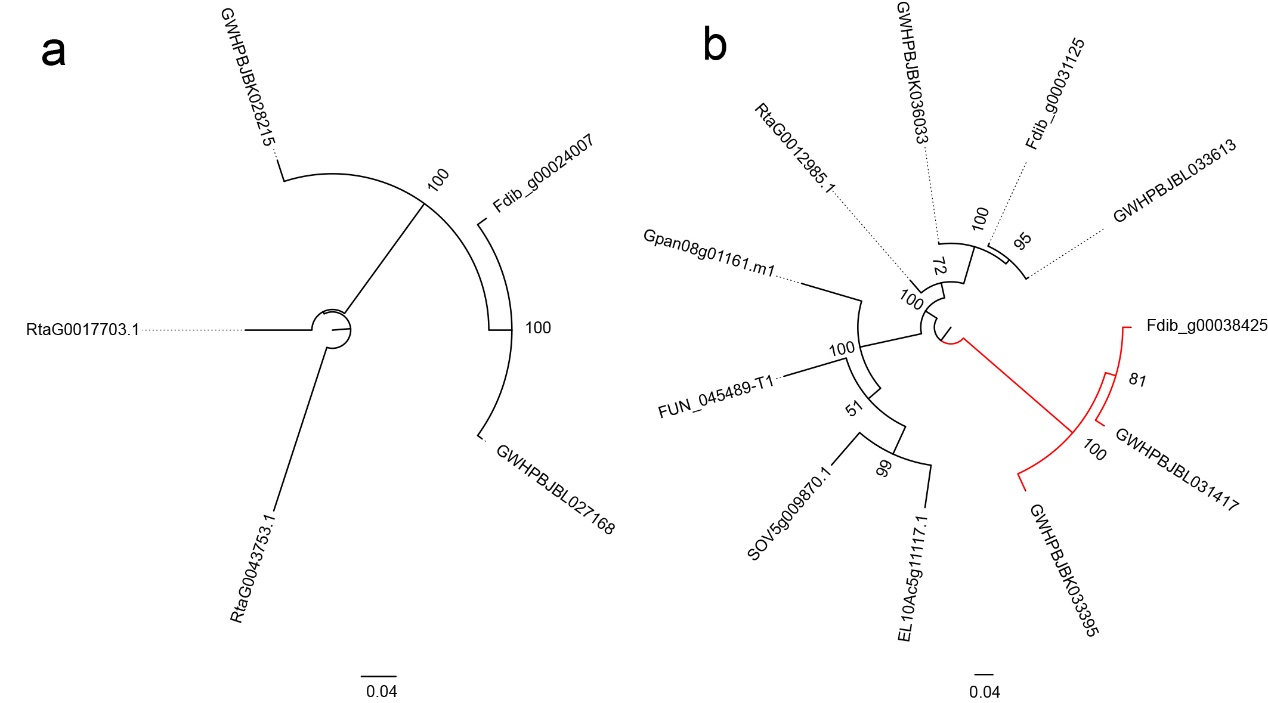


Fig S16 Phylogenetic tree of *F3'5'H* and *F3'H* gene. a: phylogenetic tree of *F3'5'H* gene; b: phylogenetic tree of *F3'H* gene, red branch represents the genes which seem to generate by gene family expansion.


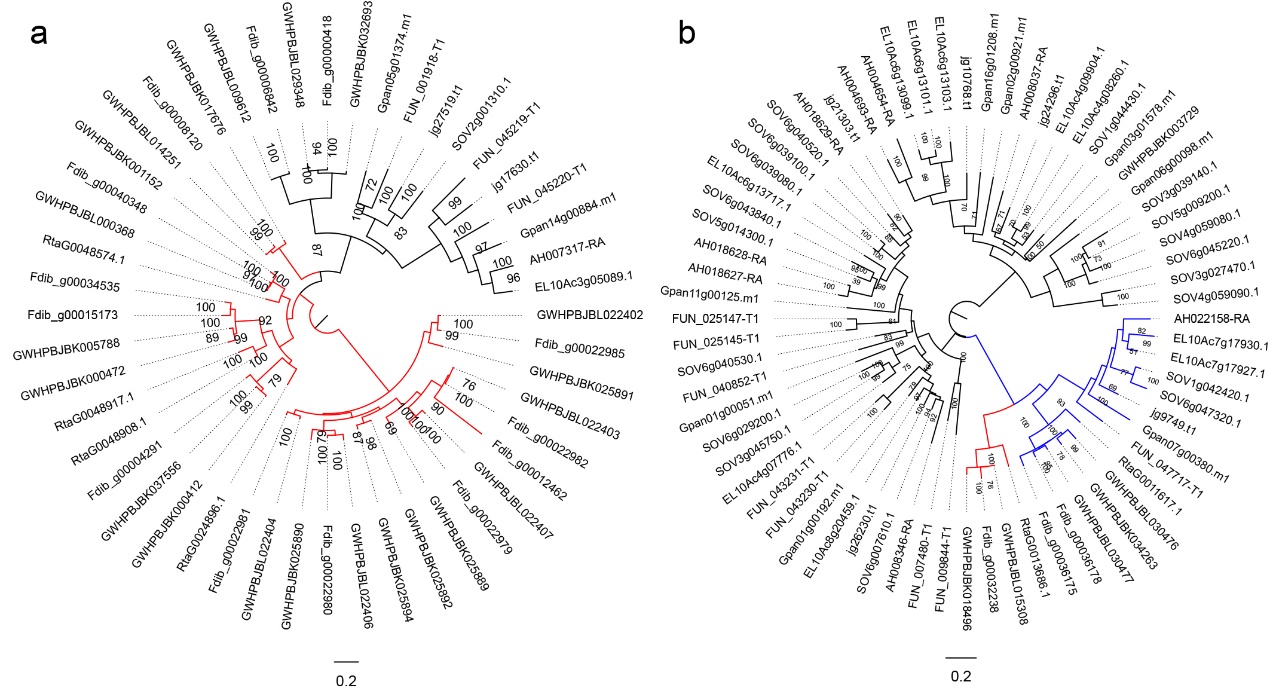


Fig S17 Phylogenetic tree of *UGT78D* and *RT* gene. a: phylogenetic tree of *UGT78D* gene, red branch represents the genes that seem to be generated by gene family expansion; b: phylogenetic tree of *RT* gene, blue branch represents the identified *RT* gene, red branch represents the genes that seem to generate by gene family expansion.


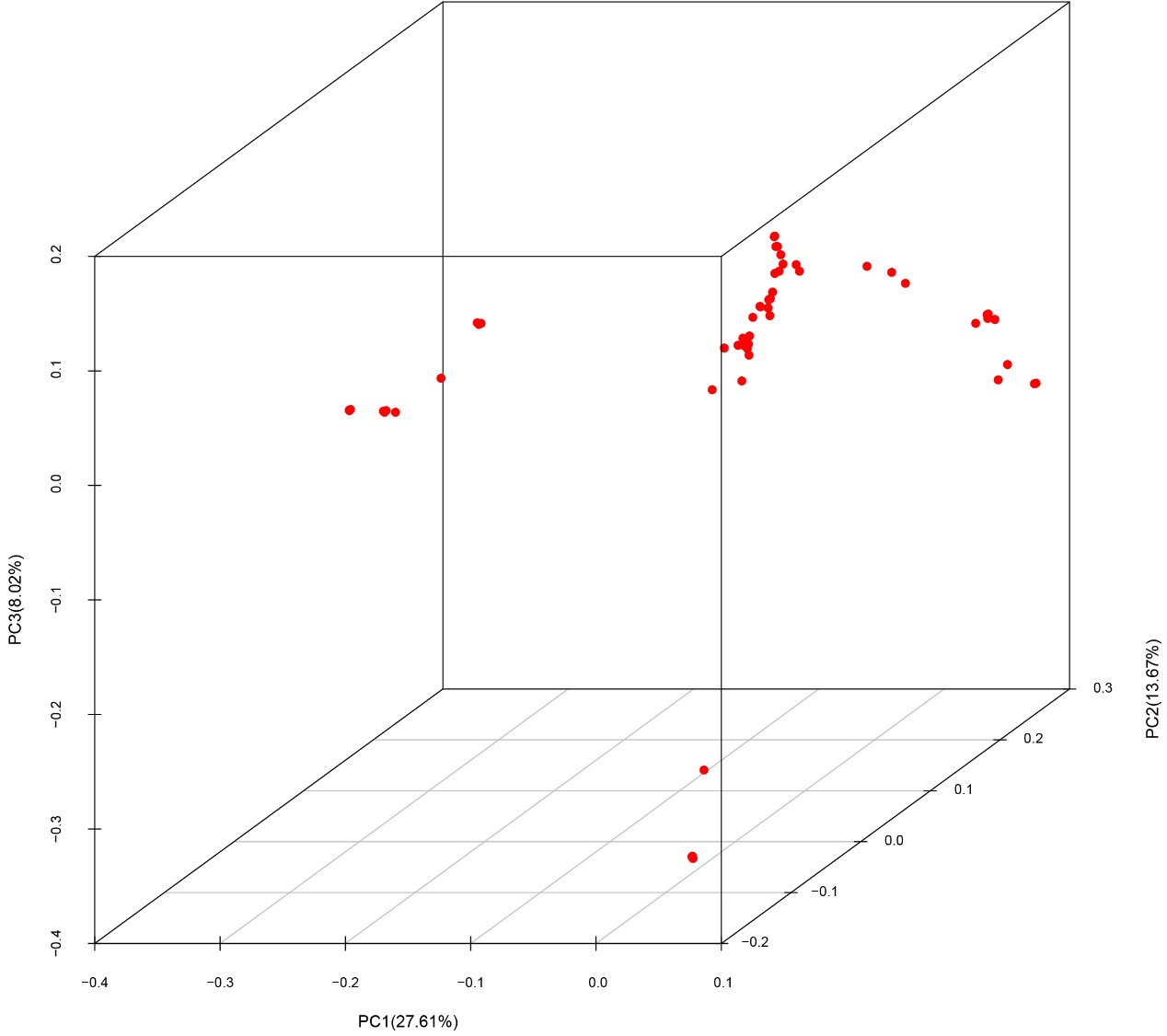


Figure S18 PCA result of resequencing, different points represent distinct resequenced individuals.


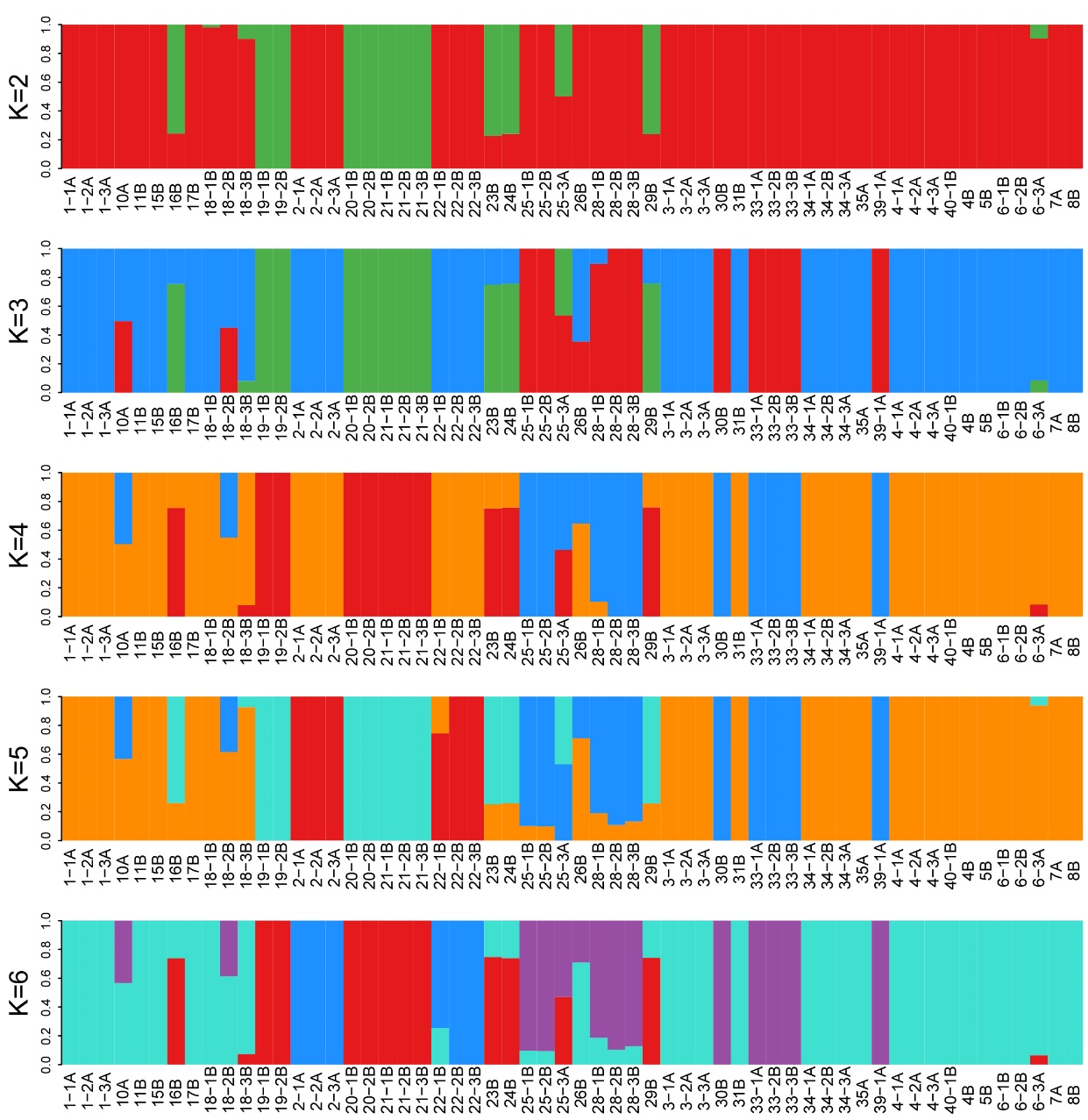


Figure S19 fastSTRUCTURE result of resequencing individuals. Different names represent different resequenced individuals.
